# Serpentinization as the source of energy, electrons, organics, catalysts, nutrients and pH gradients for the origin of LUCA and life

**DOI:** 10.3389/fmicb.2023.1257597

**Published:** 2023-10-02

**Authors:** Loraine Schwander, Max Brabender, Natalia Mrnjavac, Jessica L. E. Wimmer, Martina Preiner, William F. Martin

**Affiliations:** ^1^Institute of Molecular Evolution, Biology Department, Math. -Nat. Faculty, Heinrich-Heine-Universität, Düsseldorf, Germany; ^2^Microcosm Earth Center, Max Planck Institute for Terrestrial Microbiology and Philipps-Universität, Marburg, Germany

**Keywords:** serpentinization, hydrothermal vents, origin of life, microbial metabolism, astrobiology, hydrogen, methane, Lost City

## Abstract

Serpentinization in hydrothermal vents is central to some autotrophic theories for the origin of life because it generates compartments, reductants, catalysts and gradients. During the process of serpentinization, water circulates through hydrothermal systems in the crust where it oxidizes Fe (II) in ultramafic minerals to generate Fe (III) minerals and H_2_. Molecular hydrogen can, in turn, serve as a freely diffusible source of electrons for the reduction of CO_2_ to organic compounds, provided that suitable catalysts are present. Using catalysts that are naturally synthesized in hydrothermal vents during serpentinization H_2_ reduces CO_2_ to formate, acetate, pyruvate, and methane. These compounds represent the backbone of microbial carbon and energy metabolism in acetogens and methanogens, strictly anaerobic chemolithoautotrophs that use the acetyl-CoA pathway of CO_2_ fixation and that inhabit serpentinizing environments today. Serpentinization generates reduced carbon, nitrogen and — as newer findings suggest — reduced phosphorous compounds that were likely conducive to the origins process. In addition, it gives rise to inorganic microcompartments and proton gradients of the right polarity and of sufficient magnitude to support chemiosmotic ATP synthesis by the rotor-stator ATP synthase. This would help to explain why the principle of chemiosmotic energy harnessing is more conserved (older) than the machinery to generate ion gradients via pumping coupled to exergonic chemical reactions, which in the case of acetogens and methanogens involve H_2_-dependent CO_2_ reduction. Serpentinizing systems exist in terrestrial and deep ocean environments. On the early Earth they were probably more abundant than today. There is evidence that serpentinization once occurred on Mars and is likely still occurring on Saturn’s icy moon Enceladus, providing a perspective on serpentinization as a source of reductants, catalysts and chemical disequilibrium for life on other worlds.

## 1. Introduction

The question of how life arose is sometimes called, “the biggest question in science,” which is possibly an exaggeration. What is true is that it is perhaps the only scientific question to which everyone would like to know the answer. The origin of living things has concerned humans since antiquity. Modern scientific approaches to the problem build on Pasteur’s 19th century demonstration that life cannot be created spontaneously (Bulloch, 1938), except of course, at the origin of life from the elements of the early Earth, and Darwin’s inference that we all originate from a single common ancestor (Darwin, 1859). Mereschkowsky et al. (1910) proposed that the first organisms on Earth were probably thermophilic chemolithoautotrophs, a concept that — for good reasons — is still current among microbiologists, while Oparin and Haldane proposed a theory involving a primordial soup (Haldane, 1929; Oparin, 1957) of the type that Miller synthesized in the famous 1953 experiment powered by electric discharge (Miller, 1953).

Oparin and Haldane as well as Miller worked under the assumption that the early Earth had a reducing atmosphere. More recent studies indicate that early Earth’s mantle was oxidized, which in turn released oxidized compounds such as CO_2_, N_2_ and H_2_O into the atmosphere such that the early Earth’s atmosphere must have also been oxidizing (Sossi et al., 2020). However, reducing conditions are needed for the origin of life because, despite the coexistence of contradictory theories on origins, they all tend to concur that the carbon source for the first complex molecules was ultimately CO_2_, which needs reducing conditions to react to more complex organic molecules. There are currently two general categories of solutions afloat in the literature as to where to obtain reductants at origins: the surface (the atmosphere) and the subsurface (the crust via serpentinization).

In surface reduction models, additional impactors subsequent to the Moon-forming impact are evoked as a source of native metals at the surface. These additional impactors generate (theoretical) transient phases of reducing conditions in the atmosphere, phases that are argued to reduce CO_2_ and N_2_ to cyanide that is, in turn, central to some models for the origin of life (Benner et al., 2020; Zahnle et al., 2020; Itcovitz et al., 2022). Notably, the additional impactors are not required to explain the composition of the early Earth or the early atmosphere, they are solely “required” as a source of transient supplies of gas phase ammonia, cyanide and nitriles as starting materials for the laboratory-style organic synthesis of RNA bases, under the dual assumption that i) RNA arose from cyanide and nitriles on the Earth’s surface and that ii) something like an RNA world ever existed (Benner et al., 1989; Zahnle et al., 2007; Righter et al., 2008; Sleep, 2016; Sasselov et al., 2020; Grewal et al., 2023). Surface reduction models do not interface well with microbial metabolism, because there are neither cyanides nor nitriles in the biosynthetic pathways to bases, amino acids and cofactors and no microbe is known to require cyanide as a growth substrate. Geochemical sources of cyanide are not known, not even from volcanic exhalates (Rose et al., 2006).

In subsurface reduction models, the source of reductant for origins is geochemical and continuous: H_2_ supplied by serpentinization. The electrons released by serpentinization reduce H_2_O to H_2_ which in turn reduces CO_2_ to CH_4_ and organics (Sleep et al., 2004; Martin et al., 2008; Sleep et al., 2011; McCollom and Seewald, 2013). The diffusible reductant, H_2_, is generated within the crust, where suitable catalysts for CO_2_ reduction such as awaruite (Ni_3_Fe) and magnetite (Fe_3_O_4_) are synthesized (Preiner et al., 2018). If life originated in serpentinizing systems, it would not have needed anything from the Earth’s early atmosphere except N_2_ and CO_2_, because serpentinizing systems create the reducing conditions essential for the origin of life (Russell et al., 2010). The initial products of H_2_ dependent CO_2_ reduction under serpentinizing conditions in the laboratory are formate, acetate, pyruvate and methane, which comprise the backbone of microbial carbon and energy metabolism in organisms that use the acetyl-CoA pathway (Preiner et al., 2020). Subsurface reduction models have good congruence with microbial carbon and energy metabolism. Nucleic acid synthesis that might underpin an RNA world is more challenging under hydrothermal vent conditions (Muchowska et al., 2020; Yi et al., 2022; Harrison et al., 2023) but at the same time, there is no clear evidence that an RNA world ever existed (Baross and Martin, 2015) and there are no self-replicating RNA molecules in any life form as far as we know. Here we will focus on subsurface reduction as it occurs in modern serpentinizing systems as a starting point for origins.

Isotope evidence suggests that the first forms of life existed at least 3.8 billion years ago because carbon with an isotope signature lighter than that of abiogenic reduced carbon appears in sediments of that age (Mojzsis et al., 1996). Such light isotope signatures (δ^13^C) in the range of –40‰ to –80‰ are generally interpreted to indicate the presence of methanogens (archaea) (Arndt and Nisbet, 2012), but acetogens (bacteria) have a similarly light isotopic signature (Blaser et al., 2013). Ultralight isotopes indicate the presence of the acetyl-CoA pathway of CO_2_ fixation in primordial bacteria and archaea (Tashiro et al., 2017), in line with its exergonic nature (Berg, 2011), ancient physiology (Rühlemann et al., 1985; Fuchs, 2011), abundance of metal cofactors (Martin and Russell, 2003; Ragsdale, 2006) and carbon-metal bonds (Martin, 2020), its dual role as a pathway of carbon and energy metabolism in acetogens and methanogens (Martin and Russell, 2007) and in line with metabolic and phylogenetic reconstructions of LUCA (Weiss et al., 2016).

Methanogens and acetogens are chemolithoautotrophs and therefore grow anywhere where sufficient H_2_ and CO_2_ exists and where temperatures are biocompatible (Thauer et al., 2008; Schuchmann and Müller, 2014), from rumen to termite guts to anaerobic environments such as hydrothermal vents on the sea floor. Deep-sea hydrothermal vents were first discovered in 1977 near the Galapagos islands (Corliss et al., 1979) and since then they have been of interest for theories on the origin of life (Corliss et al., 1981; Baross and Hoffman, 1985). Criticism of vents as sites for the origin of life arose very quickly, however, because of the high temperature of black smokers (*ca.* 400°C) (Bada and Lazcano, 2002): the upper limit for life is currently 122°C (Takai et al., 2008). This is one reason why it was proposed that if life originated at a hydrothermal vent, it must have happened at a low-temperature, alkaline hydrothermal vent that undergoes serpentinization (Russell et al., 1994, 1997), a proposal that predated the discovery of the Lost City hydrothermal field (Kelley et al., 2001, 2005; Proskurowski et al., 2008) which contains multiple serpentinizing deep-sea alkaline hydrothermal vents. The Lost City hydrothermal field offers a window in time for the study of the origin of life (Martin and Russell, 2007) and fosters ideas for laboratory experiments that simulate the reducing and catalyst rich conditions of hydrothermal vents (McCollom, 2013; McCollom and Seewald, 2013; Schrenk et al., 2013; Möller et al., 2017; Muchowska et al., 2017; Boyd et al., 2020; Preiner et al., 2020).

Serpentinization releases energy, generates reductants, and provides small organic compounds that directly interface with microbial metabolism. It occurs both in terrestrial systems (continental, on land) and in submarine systems on the sea floor, usually close to the borders of tectonic plates (Schrenk et al., 2013; Wang et al., 2014; Preiner et al., 2018). Continental serpentinizing systems, for example those hosted by ophiolites, are a valuable source of information about the process, as deep-sea hydrothermal vents are much harder to access and few Lost City type systems have been discovered so far (Lecoeuvre et al., 2021). Even more remote are, of course, environments outside Earth, but they are relevant in an astrobiology context, where serpentinization has come into focus as a source of energy and reductant. There is accumulating evidence to suggest that serpentinization has occurred on Mars and might still be occurring on Mars and Enceladus (a moon of Saturn). It is suspected to be an important process on other icy moons of the gas giants in our solar system, especially where their oceans have contact with the rocky core, such as Europa and Titan (Schulte et al., 2006; Zolotov, 2007; Ehlmann et al., 2010; Glein et al., 2015; Vance et al., 2016; Waite et al., 2017; Johnson et al., 2019; Steele et al., 2022). It is useful to collate and compare data from different serpentinizing systems, which will be shown in section 3.

## 2. The importance of gradients and catalysts

When the effluent of submarine hydrothermal vents interfaces with seawater at the ocean floor, two fluids with different physicochemical properties continuously mix, generating far from equilibrium conditions over the lifespan of the vent, which in the case of Lost City can be over 30,000 years (Früh-Green et al., 2003). Some very hot hydrothermal fields can also reach ages in excess of 30,000 years (Kuznetsov et al., 2006) but this does not necessarily describe the age of individual active chimneys. The continuous far from equilibrium state of serpentinizing systems produces pH-, temperature- and redox-gradients, which create a steady supply of chemical energy sources that are similar to those used by modern microbes to run metabolic reactions and synthesize ATP. The gradients at hydrothermal vents were recognized early on as harboring similarity to energy releasing processes of cells, providing links between chemical processes in the early Earth and early forms of life and hence conducive to origin of life processes (Corliss et al., 1981; Baross and Hoffman, 1985; Holm, 1992; Russell and Hall, 1997). Gradients are interesting in an origins context because all living cells generate gradients during life and growth.

Temperature gradients at hydrothermal vents have been extensively studied by Braun and coworkers in the context of thermophoresis, a physicochemical process that leads to chemical concentration gradients in physically confined compartments (Baaske et al., 2007; Möller et al., 2017; Hudson et al., 2020; Matreux et al., 2023). Thermophoresis can lead to orders of magnitude accumulation of organic compounds in compartments on scales the size of μm to cm. All theories for origins require sufficient concentrations of chemical reactants in order to react at a significant rate, generating enough products to react further and attain higher complexity. Naturally occurring networks of inorganic microcompartments that form at the vent-ocean interface of serpentinizing hydrothermal systems can serve as sites of natural chemical concentration processes (Russell and Hall, 1997; Martin and Russell, 2003) thermophoresis amplifies that effect by orders of magnitude (Baaske et al., 2007).

The pH gradients of serpentinizing systems are important in an origins context for two reasons. First, the alkaline nature of effluent in serpentinizing systems, in the range of pH 9–11, stemming from continuous Mg (OH)_2_ synthesis during the serpentinization process (Chavagnac et al., 2013), together with high H_2_ concentrations of 1–10 mM or more, generates extremely negative redox potentials, on the order of –435 to –830 mV which where both measured and calculated at and for serpentinizing systems (Suzuki et al., 2017; Boyd et al., 2020; Nobu et al., 2023; Quéméneur et al., 2023). Provided that enough catalysts are present (Preiner et al., 2018) these conditions are sufficient to abiotically reduce CO_2_ (or solid phase inorganic carbon) to formate, with the result that formate of abiotic origin is a very common solute in the effluent of serpentinizing systems (Lang et al., 2010, 2018), continuously present in micromolar concentrations. Formate can serve as a substrate for growth of methanogens (Dolfing et al., 2008; Stams and Plugge, 2009) and acetogens (Moon et al., 2021), microbial groups that inhabit serpentinizing systems (Fones et al., 2021; Kraus et al., 2021; Lecoeuvre et al., 2021; Brazelton et al., 2022; Colman et al., 2022). The synthesis of formate in high mM amounts from H_2_ and CO_2_ is readily catalyzed by minerals such as awaruite (Ni_3_Fe), magnetite (Fe_3_O_4_) and greigite (Fe_3_S_4_) under hydrothermal conditions in the laboratory (Preiner et al., 2020).

The second role of pH gradients in serpentinizing systems is that they generate a continuous and natural chemiosmotic gradient with the same polarity as cells. Modern oceans have a pH of 7.8–8.2 […] with a calculated pH of 6.6 because of higher CO_2_ concentrations (Schrenk et al., 2013; Krissansen-Totton et al., 2018). At the vent-ocean interface, this generated a natural proton gradient of roughly three pH units, a far greater gradient than the roughly one pH unit or less that modern cells require to power their rotor-stator ATP synthetase (Tran and Unden, 1998; Silverstein, 2014). Geochemically formed pH gradients could have been harnessed by cells before the origin of proton pumping proteins, which would explain why the ATP synthase and the principle of chemiosmotic energy conservation is universally conserved across all prokaryotic cells while the proteins that generate ion gradients are not (Martin and Russell, 2007; Lane et al., 2010; Lane and Martin, 2010).

It has been suggested that pH gradients might themselves be a source of chemical energy for CO_2_ reduction (Sojo et al., 2019; Hudson et al., 2020). Hudson et al. (2020) reported *ca.* 1 μM formate in the presence of gradients, Sojo et al. (2019) obtained no gradient dependent CO_2_ reduction. A number of recent studies of H_2_-dependent CO_2_ fixation under simulated hydrothermal vent conditions clearly show that no gradient or compartmentation is required for CO_2_ reduction. Preiner et al. (2020) obtained 0.3 M formate, and up to 560 μM acetate and 10 μM pyruvate (C3 synthesis) from H_2_ and CO_2_ using alkaline vent conditions in free solution with no gradients or compartments. The key to organic synthesis in serpentinizing systems are not gradients but catalysts, for example hydrothermally formed minerals like awaruite, magnetite and greigite in the case of Preiner et al. (2020), or Ni or Fe metals (Muchowska et al., 2017). Recent work by the group of Harun Tüysüz in Mülheim has characterized in considerable detail the properties that influence the efficiency of CO_2_ reduction reactions with nanoparticular and silicate-supported solid state Fe, Ni and Co catalysts (Belthle et al., 2022; Beyazay et al., 2023a,b). For organic synthesis under simulated hydrothermal vent conditions, the catalytic properties of the solid phase and the right reactants are key, not the existence of gradients *per se*.

## 3. Serpentinizing systems and their similarities and differences

Serpentinization involves reactions of ultramafic rocks with water. Ultramafic rocks have low silicate (SiO_2_) content (<45 %) and have a relatively high magnesium and iron content (the term *mafic* stems from *ma*gnesium and *fe*rrum rich). Olivine and orthopyroxene are the main minerals of ultramafic rocks (Proskurowski et al., 2008). Serpentinization produces hydrogen gas (H_2_), which in Fischer-Tropsch type reactions (FTT) can lead to the production of methane (CH_4_) and other hydrocarbons at low temperatures (<150°C) through the reduction of carbon dioxide (CO_2_) (Etiope and Sherwood Lollar, 2013; Etiope and Schoell, 2014). The serpentinization reaction of olivine, an iron-magnesium silicate, and water to serpentinite and iron-rich brucite, with further reaction of the latter with silica to serpentinite, magnetite and hydrogen, can be written in several ways. Bach et al. (2006) summarize the reaction as:


$$\begin{array}{l} {2Mg}_{1.8}{Fe}_{0.2}{SiO}_{4}+3H_{2}O\to {\text{Mg}}_{2.85}{Fe}_{0.15}{Si}_{2}O_{5}{\left(OH\right)}_{4}+\\ \;{\text{Mg}}_{0.75}{Fe}_{0.25}{\left(OH\right)}_{2} \end{array}$$

$$\begin{array}{l} 57{\text{Mg}}_{0.75}{Fe}_{0.25}{\left(OH\right)}_{2}+30{SiO}_{2}\left(aq\right)\to \\ 15{\text{Mg}}_{2.85}{Fe}_{0.15}{Si}_{2}O_{5}{\left(OH\right)}_{4}+23H_{2}O+{4Fe}_{3}O_{4}+4H_{2} \end{array}$$

while Chamberlain et al. (1965) summarize the reaction as follows:


$$\begin{array}{l} {6Mg}_{1.5}{Fe}_{0.5}{SiO}_{4}\;\left(\text{olivine}\right)+7H_{2}O\to \\ {3Mg}_{3}{Si}_{2}O_{5}{\left(OH\right)}_{4}\;\left(\text{serpentine}\right)+{Fe}_{3}O_{4}\;\left(\text{magnetite}\right)+H_{2} \end{array}$$

whereby Sleep et al. (2011) simplify the reaction to the essential H_2_-production process:


$$\begin{array}{l} 3\text{FeO}\;\left(\text{in rock}\right)+H_{2}O\;\left(\text{liquid}\right)\to \\ H_{2}\;\left(\text{in solution}\right)+{Fe}_{3}O_{4}\;\left(\text{magnetite}\right) \end{array}$$

The H_2_ produced by serpentinization can reduce CO_2_ to organic compounds and methane, N_2_ to ammonia, and divalent Fe and Ni ions to mineral alloys of native metals. Serpentinization is an abundant source of geological reducing power. Paul Sabatier first discovered the abiotic formation of methane in 1913 by letting CO_2_ and H_2_ react with metal catalysts. Franz Fischer and Hans Tropsch generated more complex hydrocarbons with CO and H_2_ in 1925. Both are labeled Fischer-Tropsch-type (FTT) reactions (Etiope and Schoell, 2014). Large quantities of abiotic gas are generated on Earth by such reactions. As soon as a CO_2_ source is available to a serpentinizing system, FTT reactions produce CH_4_ by serpentinization (Etiope and Sherwood Lollar, 2013; Etiope and Schoell, 2014). Transition metals including Ni and Fe as well as transition metal minerals including magnetite (Fe_3_O_4_) and awaruite (Ni_3_Fe) catalyze FTT reactions. The conversion of the gas occurs after chemisorption to the metal surface (Sleep et al., 2004; Etiope and Schoell, 2014; Holm et al., 2015; Preiner et al., 2018). Ni, Fe and Cr are the most abundant metals in ultramafic rock. Both serpentinization and FTT reactions are considered to be important for origin of life because they forge C–C bonds from CO_2_ and CO as starting materials (Russell et al., 2010). In an astrobiological context, they are suspected to be involved in the production of hydrocarbons on other planets (Etiope and Schoell, 2014).

H_2_ is a source of energy and an electron donor for microorganisms in modern serpentinizing systems, and it could have carried out the same role in prebiotic chemistry (Etiope, 2017). The generation of H_2_ from serpentinization is itself exergonic and has been taking place on Earth ever since there was water present (Sleep et al., 2004; Preiner et al., 2018). On the early Earth the lithosphere was not yet differentiated and higher mantle temperatures allowed ultramafic lava to erupt on Earth’s surface, such that ultramafic rocks were probably much more exposed to the surface oceans than today, meaning that serpentinization was almost certainly much more prevalent on early Earth than now (Sleep et al., 2004, 2011; McCollom and Seewald, 2013; Schrenk et al., 2013). Serpentinization can occur at temperatures lower than <100°C (Holm et al., 2015) and usually generates an alkaline pH, especially at lower temperatures (usually > pH 9) because protons are consumed and free OH^–^ is released during the process that leads to the production of H_2_ (Mottl et al., 2003; Schrenk et al., 2013).

Products of serpentinization generate […] formate and CH_4_ as the main products of CO_2_ reduction measured in modern vents. Methane is always present in continental serpentinization sites and usually in considerable amounts. Lang et al. (2010) reported acetate in the effluent of Lost City but suggested that it stemmed from microbial metabolism. Abiotically formed acetate has not been detected in the effluent of serpentinizing hydrothermal systems to our knowledge. A recent report detected 1.2 mM abiotic acetate in host rocks of the Canadian Shield (Sherwood Lollar et al., 2021), although there is no direct evidence of it being synthesized by serpentinization so far. In effluent samples, CH_4_ concentrations range from 0.01 to 14 mg/L, while normal water in equilibrium with the atmosphere has a CH_4_ concentration of roughly 0.00003 mg/L. Longer-chain hydrocarbons (ethane to pentane) are also abiotically produced in serpentinizing systems, both submarine (Proskurowski et al., 2008) and on the surface (Etiope and Schoell, 2014), however in much lower quantities than methane.

Continental serpentinization sites have been studied since at least the 1960s (Barnes et al., 1967) and some of them, such as Chimera in Turkey, have been known to humans for thousands of years (Etiope, 2015). There are three types of mafic host rocks that support continental serpentinizing systems: ophiolites (ultramafic mantle rocks obducted to the surface), orogenic peridotite massifs and igneous intrusions. Some continental serpentinizing systems have been well-studied with respect to microbiology, gas composition and geology. In studies of gas composition, the isotope compositions of CH_4_ are particularly important to determine whether it has a biogenic or abiogenic origin (Etiope and Schoell, 2014). Differentiating the two is difficult because the gases in these systems can be a mix of both abiotic and biotic gas, as for example, in The Cedars spring in California, where abiotic gas is present, but microbes also produce CH_4_ through CO_2_ reduction because of the abundant H_2_ present as their energy source (Nealson, 2005; Morrill et al., 2013; Etiope and Schoell, 2014; Suzuki et al., 2017).

### 3.1. Hydrothermal vent types

**Figure 1:**
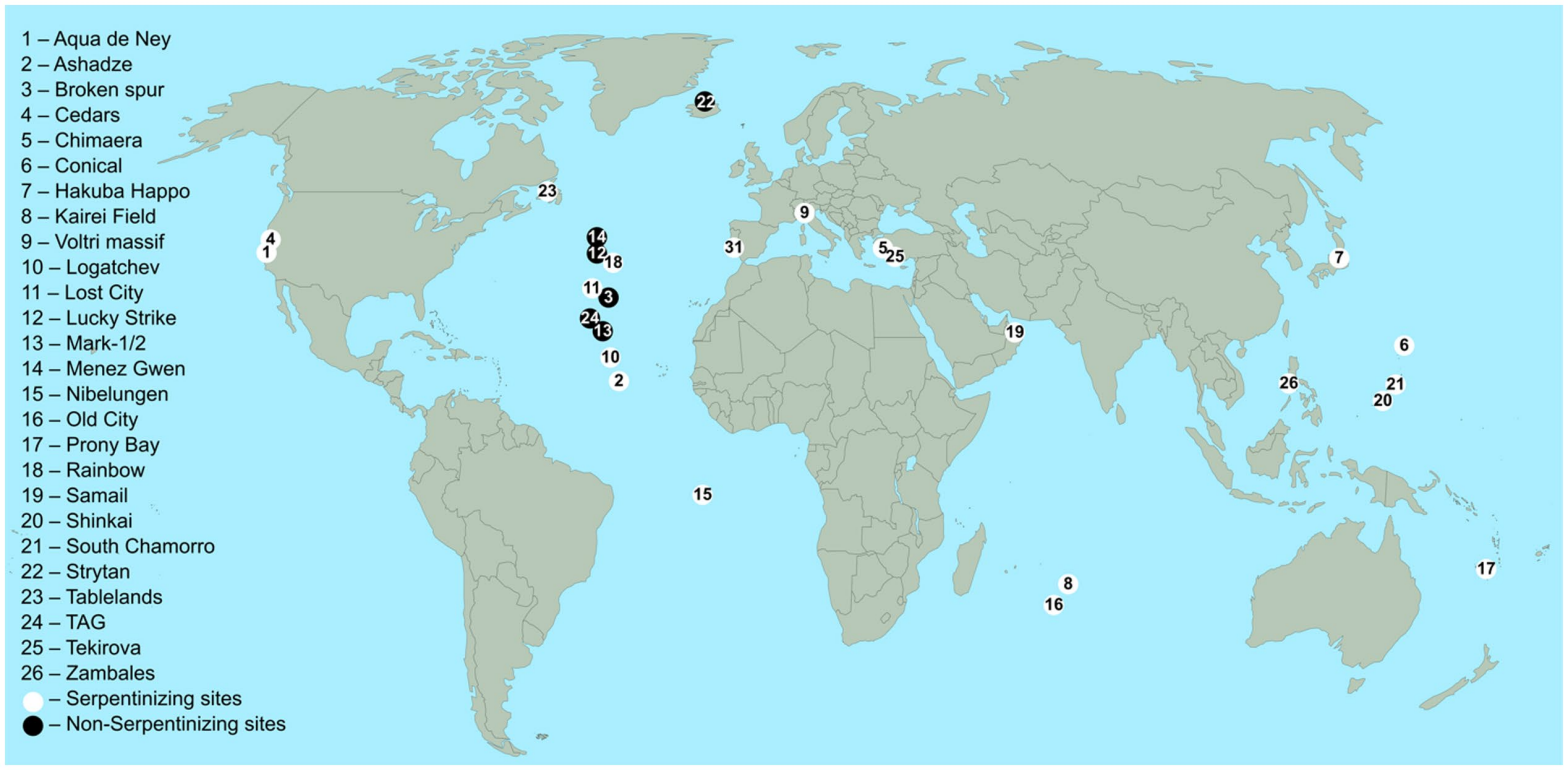
Locations of the serpentinizing and basalt hosted sites listed in Table 1. White circles indicate serpentinizing sites, black circles indicate non-serpentinizing sites. The sample of studies shown is not comprehensive and makes no statement about the relative frequencies of serpentinizing vs. non-serpentinizing sites. References: See Table 1.

Hydrothermal vents are often divided into two types — black and white smokers — but off ridge systems like Lost City belong to neither category because they do not emit “smoke.” Both the smoke of black and white smokers comes from metal sulfide and calcium and barium precipitation, but white smokers vent slower and at slightly lower temperatures (220–330°C) than black smokers, which is why a greater percentage of the metals precipitates in the chimneys and is not vented out causing the white color of the smoke, Lost City type vents have carbonate chimneys but no smoke (Rona et al., 1986; Kelley et al., 2005). A map showing the geographical distribution of some vents discussed here is shown in Figure 1.

Black smokers are hydrothermal fumaroles with abundant sulfides usually located close to or directly on top of the spreading zones of mid-ocean ridges. Because of their proximity to magma, the water in them can be up to over 400°C hot. They are also characterized by a low pH, even though black smokers that are hosted in ultramafic rocks can host serpentinization, such as the Rainbow and Logatchev hydrothermal fields on the mid Atlantic ridge (MAR) (Schrenk et al., 2013). These serpentinizing black smokers, in contrast to Lost City type vents, have higher temperatures, higher metal concentrations and an acidic pH (3–4), but unlike hydrothermal vents hosted in basaltic rock, they have high hydrogen and methane concentrations like other serpentinizing systems. The Rainbow and Logatchev systems are the best studied vents of this type. They also vent C2–C5 hydrocarbons in trace amounts (Charlou et al., 2002; Schrenk et al., 2013). Serpentinization does not happen in basalt-hosted vents because of the higher silica content of the basaltic rock (McCollom and Seewald, 2013).

Off ridge vents, or Lost City type vents, are characterized by carbonate chimneys, a high effluent pH (9 to 11) and moderate temperatures (between 28 to 116°C) compared to black or white smokers (Proskurowski et al., 2008; Seyfried et al., 2015). They are mainly represented by the first system of its kind discovered: the Lost City hydrothermal field. Lost City is located 750 m below sea level, but 4 km above the sea floor on the Atlantis massif (Kelley et al., 2005). Submarine systems that are comparable to Lost City are Prony Bay in New Caledonia (Monnin et al., 2014), the Old City hydrothermal field in the Indian ocean (Lecoeuvre et al., 2021) and the Shinkai Seep Field on the Mariana arc (Okumura et al., 2016).

Prony Bay is in much shallower seawater than Lost City (only 16 to 47 m below the surface) and its water source is fresh water, not crustal circulated seawater as in Lost City (Kelley et al., 2001; Quéméneur et al., 2014; Postec et al., 2015; Twing et al., 2022). Other important differences between shallow seawater vents and deep-sea vents are the access to sunlight, the influence of tidal forces and the different pressures (Price and Giovannelli, 2017). However, in terms of effluent parameters, Prony Bay is more similar to Lost City than the black smokers or the continental serpentinizing systems. It has elevated concentrations of H_2_ and CH_4_, a high pH and low venting temperatures (see Table 1) (Monnin et al., 2014).

**Table 1:** Parameters of different serpentinizing and basalt-hosted systems covered in this review.

Name	Loc/Water/Serp	Temp [°C]	Depth [m]	pH	H_2_	CH_4_	CO_2_
Aqua de Ney^1^	USA / f / Y	11.6	sur.	13	n.d.	1.92 mM	n.d.
Ashadze^2,3^	MAR / s / Y	296–372	–	3–4	19–26.5 mM	0.8–1.2 mM	n.d.–3.7 mM
Broken spur^4^	MAR / s / N	356–364	3,200	–	0.43–1.03 mM	65–130 μM	6.0–7.1 mM
Cedars^1^	USA / f / Y	17.4	sur.	12	157 μM	22 μM	44 μM
Chimaera^5^	Turkey / f / Y	–	sur.	–	9.82 vol%	85.33 vol%	trace
Conical^2^	MF / s / Y	–	3,083	12.5	–	2 mM	–
Hakuba Happo^6,7^	Japan / f / Y	50–60	500 (terr.)	>10	201–664 μM	124–201 μM	n.d.
Kairei Field^2^	CIR / s / Y	–	2,400	–	8 mM	500 μM	–
Voltri massif^2^	Italy / f / Y	10.5–23	sur.	10–12	–	6–867 μM	12–3,636 μM
Logatchev^2-4,8^	MAR / s / Y	320–352	3,000	3–4	11.1–12.5 mM	1.2–2.6 mM	4.4–10.1 mM
Lost City^2,3,9-11^	MAR / s / Y	40–116	7–800	9–12	0.5–16 mM	1–2 mM	trace
Lucky Strike^4^	MAR / s / N	170–364	1700	3.5	20–730 μM	0.5–0.97 mM	13–28 mM
Mark–1/2^4^	MAR / s / N	335–350	3,460	4	190–480 μM	23–62 μM	5.2–6.7 mM
Menez Gwen^4^	MAR / s / N	275–284	850	4.3	24–48 μM	1.35–2.63 mM	17–20 mM
Nibelungen^2^	MAR / s / Y	192–372	3,000	3	11.4 mM	1.4 mM	–
Old City^12,13^	CIR / s / Y	–	3,100	8	–	–	–
Prony Bay^2,14-16,22^	N.C. / f + s / Y	22–43	16–47	8–11	21.1–731.3 μM	153–376.6 μM	trace
Rainbow^4^	MAR / s / Y	365	2,300	3	12.9–16 mM	1.65–2.5 mM	16–17 mM
Samail^2,17^	Oman / f / Y	25–36.3	sur.	9–12	253 μM	0.1–483 μM	n.d.
Shinkai^18^	MF / s / Y	–	5,555	–	–	–	–
S. Chamorro^2^	MF / s / Y	–	2,960	12.5	–	2 mM	–
Strytan^13,19^	Iceland / f / N	70	16–70	<10	0.1–5.2 μM	0.5–1.4 μM	–
Tablelands^1,2^	Canada / f / Y	9.6	sur.	12–13	30–600 μM	23.7 μM	25–619 μM
TAG^4^	MAR / s / N	290–321	3,670	3	150–370 μM	124–147 μM	2.9–3.4 mM
Tekirova^2,5,20^	Turkey / f / Y	–	–	–	7.5–11.3 vol%	65–93 vol%	trace
Zambales^2,20,21^	Phillip. / f / Y	110–125	sur.	–	8.4–45.6 vol%	13–55 vol%	trace

Loc = Location; Water = Water source; f = freshwater source; s = seawater source; Serp = Serpentinizing system; Y = Yes (serpentinizing system); N = No (non-serpentinizing system, basalt hosted); MAR = Mid Atlantic Ridge = Atlantic Ocean; MF = Mariana Forearc, Pacific Ocean; CIR, Central Indian Ridge, Indian Ocean; N.C. = New Caledonia; Phillip = Phillippines; sur, surface = continental serpentinizing site; terr = terrestrial site, a hole was drilled 500 m into the ground; n.d. = not detected; trace = trace amounts detected; –, not reported.

^1^(Cook et al., 2021), ^2^(Schrenk et al., 2013), ^3^(Konn et al., 2015), ^4^(Charlou et al., 2002), ^5^(Etiope et al., 2011b), ^6^(Suda et al., 2017), ^7^(Suda et al., 2014), ^8^(Proskurowski et al., 2008), ^9^(Kelley et al., 2001), ^10^(Kelley et al., 2005), ^11^(Seyfried et al., 2015), ^12^(Lecoeuvre et al., 2021), ^13^(Twing et al., 2022), ^14^(Monnin et al., 2014), ^15^(Postec et al., 2015), ^16^(Quéméneur et al., 2014), ^17^(Kraus et al., 2021), ^18^(Okumura et al., 2016), ^19^(Price and Giovannelli, 2017), ^20^(McCollom and Seewald, 2013), ^21^(Abrajano et al., 1988), ^22^(Quéméneur et al., 2023).

The main difference in effluent chemical composition between the high temperature black smokers that serpentinize like the Rainbow and Logatchev hydrothermal fields and the low temperature Lost City hydrothermal field, is that Rainbow and Logatchev have higher concentrations of CO_2_ and metals and acidic pH in their effluent whereas Lost City has a high pH and is depleted in metals and CO_2_ (Proskurowski et al., 2008).

### 3.2. Conditions in hydrothermal vents

Continental serpentinizing systems have comparatively cool effluent under 100°C, and they also have hyperalkaline waters, although they are fed by fresh water (usually called meteoritic water, which is a complicated name for rainwater or snow) rather than altered sea water. Numerous continental serpentinization sites are known (Etiope et al., 2011b; Etiope and Schoell, 2014; Etiope, 2015). They are important analog sites to deep-sea serpentinizing sites because of their chemical similarities, and because they are easier to access. Like submarine serpentinizing systems, they also produce large amounts of H_2_ and CH_4_. They are the main source for abiotic gas on Earth (Etiope, 2017). Abiotic volatile hydrocarbons are still poorly understood but they can be generated by various inorganic mechanisms, including FTT. At some sites the amount of abiogenic methane can reach up to 90 vol% of the gas content (Etiope and Schoell, 2014), most of the remainder being H_2_.

Abiogenic methane is widely discussed in the search for extraterrestrial life because methane is considered a potential biomarker, the sign of methanogenesis. But if Earth can generate large amounts of methane through abiotic processes, other wet, rocky planets and moons probably can as well, making methane itself problematic as a sign of life (Etiope, 2017). However, if biochemistry and life arose from exergonic H_2_-dependent CO_2_ reducing reactions, then abiotic methane serves as a proxy for the existence of geochemical conditions that are conducive to life’s origin: exergonic reactions of H_2_ and CO_2_ under continuously far from equilibrium conditions.

The distinction between biotic and abiotic methane in usually made by isotope fractionation (Etiope et al., 2013; Etiope and Schoell, 2014). Abiotic methane is enriched in ^13^C relative to biotic methane, which is isotopically light because of isotope discrimination in favor of ^12^C in the acetyl-CoA pathway (Blaser et al., 2013) of methyl synthesis from H_2_ and CO_2_ underpinning methanogenesis. However distinguishing biotic and abiotic methane by carbon isotope fractionation is not always straightforward (Horita and Berndt, 1999), which is why deuterium fractionation is also often taken into account (Etiope et al., 2011a) and often even combined (Miller et al., 2018) as shown in a study conducted in the Samail Ophiolite where it could be determined that biotic and abiotic methane mix (Nothaft et al., 2021).

Radiocarbon methods (^14^C) can also be used to determine the source of C in CH_4_ from serpentinizing systems. In the upper atmosphere, ^14^C is constantly produced by neutron capture of ^14^N to generate ^14^C and a proton. The neutrons stem from cosmic radiation, the resulting ^14^C has a half-life of 5,700 years. There is no radiocarbon in CH_4_ of Lost City effluent. That is, CH_4_ from Lost City is “radiocarbon dead,” meaning that the carbon source cannot be bicarbonate from seawater, unless the circulation time exceeds 57,000 years (ten ^14^C half-lives) because seawater contains ^14^C that is constantly synthesized in the upper atmosphere. It is estimated that the entire volume of the ocean circulates through hydrothermal vents every 100,000–500,000 years (Fisher, 2005). The low CO_2_ concentrations at Lost City together with its “radio-carbon-dead” CH_4_ suggest that CH_4_ in Lost City is likely derived from CO_2_ of mantle origin that was reduced during serpentinization (Proskurowski et al., 2008).

### 3.3. CO_2_ and H_2_

Most of the carbon emitted from Lost City comes from the Earth’s mantle in the form of formate (Lang et al., 2012, 2018). Formate can be used as a substrate for methanogenesis in environments where CO_2_ is limiting. Methanogens can readily convert formate into CO_2_ and H_2_ or use it to reduce coenzyme F_420_ to F_420_H_2_, a flavin similar to FAD (Holden and Sistu, 2023). This allows them to access formate as a source of carbon and electrons for methanogenesis. This has been observed not only in Lost City but also in the Samail ophiolite (Fones et al., 2021). Formate forms abiotically under the high pH, reducing conditions of serpentinizing fluids. This can also be replicated in the laboratory (Preiner et al., 2020), where formate is typically the most abundant reduced carbon species under a variety of conditions (Belthle et al., 2022; Beyazay et al., 2023a,b). In Lost City, formate is the second most abundant carbon species after CH_4_ and the second most available reductant after H_2_ (Lang and Brazelton, 2020; Brazelton et al., 2022).

High concentrations of effluent H_2_ are characteristic for serpentinizing systems, as summarized in Table 1. In terms of H_2_ concentration, the non-serpentinizing Strytan hydrothermal field has the lowest out of all the systems where H_2_ was detected. It is in the range of normal seawater. At first glance it might seem as if continental serpentinizing systems do not have high concentrations of H_2_, however it should be mentioned that sometimes only the liquid phase was measured, not the actual gas phase emitted from the systems. In the Tekovira and Zambales ophiolite, where the gas phase composition was measured, H_2_ can comprise 45 vol% of the emitted gas. High H_2_ tends to correlate with high CH_4_, but not strictly (see Supplementary Figure S1). Lost City and other serpentinizing systems of the mid Atlantic ridge (MAR) have high concentrations of CH_4_ and H_2_. The effluent at Prony Bay, a shallow sea vent with a fresh water source and similar microbial communities as continental ophiolites, has lower H_2_ and CH_4_ concentrations than Lost City of about a magnitude but still in significant amounts (Quéméneur et al., 2023). The Ashadze system on the MAR has very high H_2_ concentration in its venting fluids of up to 26.5 mM. For CH_4_ concentrations, many deep-sea hydrothermal vents, with a few exceptions, have a concentration of 1–3 mM. In continental serpentinizing sites CH_4_ can reach between 20 vol% in New Zealand to 93 vol% of the gas phase in the Tekovira ophiolite (Etiope and Schoell, 2014). CH_4_ is also extensively studied in continental serpentinization sites because they are the main contributor to abiotic gas (Etiope, 2017).

Alkaline serpentinizing systems are low in CO_2_ because at high pH dissolved CO_2_ turns into carbonate (CO_3_^2–^), which precipitate within the vent as Ca^2+^ and Mg^2+^ carbonates. This is the reason why hot and acidic hydrothermal vents such as Rainbow or Lucky Strike have very high CO_2_ concentrations (up to 28 mM), while alkaline systems such as Lost City and Prony Bay harbor only have trace amounts of dissolved inorganic carbon. For continental serpentinization sites CO_2_ was only detected in trace amounts in the gas phase. In the liquid phase CO_2_ [often in the form of bicarbonate (HCO_3_^–^)] could sometimes be detected up to the mM range, but trace amounts are typical (Table 1). More complex organics than CH_4_, when reported, were only detected in trace amounts both in ocean and continental serpentinization systems. Formate and acetate, when reported, were detected in Lost City in μM amounts and in the Conical Seamount of the Mariana Forearc in mM (formate) and μM (acetate) amounts, whereby the acetate at Lost City is likely biogenic (Lang et al., 2010).

Comparing different serpentinizing systems across studies is not straightforward because the sampling methods are often different and because of local heterogeneity: even in the same system values can differ greatly depending on where in the system the samples were taken. Cook et al. (2021) showed that the values measured in different samples, be it the temperature, the pH or the concentrations of the different compounds, can be vastly different depending on the sampling site. For example, if the groundwater, the well water or the surface water were sampled at three different sites — Tablelands, the Cedars, Aqua de Ney — the pH values could range between pH 8–9 in the surface water to pH 12–13 in the well water.

## 4. Microbiota of hydrothermal vents: a window into the past

Serpentinizing hydrothermal vents yield insights into primordial geochemical conditions, thereby providing a window in time into the nature of the environments that the first prokaryotic communities on the early Earth inhabited and the role of inorganic catalysts in the chemical setting of life’s origin (Corliss et al., 1981; Baross and Hoffman, 1985). Serpentinizing systems also link the geochemistry of primitive environments to autotrophic theories for origins. It is estimated that about 60 % of all microbes inhabit Earth’s crust: ~35 % in the submarine crust and ~25 % in the continental crust (Flemming and Wuertz, 2019). There is no sunlight, so primary production in the crust has to start with chemolithoautotrophs. The most prevalent (and obvious) source of energy for autotrophy is the H_2_-CO_2_ couple that fuels acetogen and methanogen metabolism. Serpentinizing hydrothermal vents constantly generate H_2_ and reducing conditions that mobilize nutrients.

Many reports have investigated the chemistry and microbial communities of hydrothermal vents, earlier reviews are provided by (Kelley et al., 2002; Martin et al., 2008; Lang and Brazelton, 2020). Serpentinizing hydrothermal systems were the topic of a recent special dedicated issue of the Philosophical Transactions of the Royal Society with contributions covering new insights from the serpentinite reactions at the Marianas trench (Fryer et al., 2020), the effect of pH on H_2_ production (McCollom et al., 2020), hydrocarbons in fluid inclusions within olivine rich rocks (Grozeva et al., 2020), the role of serpentinite in the search for life beyond Earth (Vance and Daswani, 2020), the thermodynamics of the H_2_-rich environments of serpentinizing systems and how that effects microbial CO_2_ fixation (Boyd et al., 2020), a thorough review of microbial investigations at Lost City (Lang and Brazelton, 2020). Another worthy read is an in-depth study focusing on Prony Bay showing the relationship of microbial communities in hyperalkaline waters with high concentrations of H_2_ (Quéméneur et al., 2023). A number of new reports shed light on the relationship between geochemical reactions at hydrothermal vents and autotrophic origins of microbial metabolism and life. In an origins context we can focus on anaerobic processes.

### 4.1. Carbon

Although CO_2_ was very abundant on the early Earth (Sossi et al., 2020), modern serpentinizing systems are generally low in CO_2_ because of magnesium carbonate and calcium carbonate precipitation at high pH. The effluent of serpentinizing systems can however be rich in formate (HCOO^–^). Formate is readily converted by simple inorganic catalysts (Preiner et al., 2020; Belthle et al., 2022; Beyazay et al., 2023a,b) and by microbes into H_2_ and CO_2_ via the near-equilibrium reaction:


$${\text{HCOO}}^{-}+H+\to H_{2}+{CO}_{2}$$

with Δ*G*_o_’ = –3.5 kJ·mol^–1^ (Maden, 2000). Microbes have a number of different enzymes for that reaction. The formate to CO_2_ converting reaction is catalyzed by the enzyme hydrogen-dependent CO_2_ reductase, HDCR (Schuchmann and Müller, 2013), or by NADH-dependent formate dehydrogenase (FDH), which is the typical enzyme of the acetyl-CoA pathway in acetogens (Schuchmann and Müller, 2013). In methanogens the reaction is catalyzed via hydrogenases that convert H_2_ into reduced ferredoxin and the activity of formyl-methanofuran dehydrogenase, which contains a FDH domain and 46 [4Fe4S] clusters (Wagner et al., 2016). A third kind of formate dehydrogenase, HylABC-Fdh2, performs a reaction called electron bifurcation in which an electron pair is split, with the individual electrons being transferred to different acceptors with different redox potentials (Buckel and Thauer, 2013; Buckel and Thauer, 2018). HylABC-Fdh2 converts formate into CO_2_ with one of the electrons being transferred to NAD^+^ and the other being transferred to ferredoxin (Wang et al., 2013). Oxidation of two formate molecules via this reaction generates two CO_2_, one NADH and two reduced ferredoxins. This bifurcating form of FDH is found in *Bipolaricaulota*, a deeply branching acetogen identified by metagenomics in the effluent of the hyperalkaline water of the Oman ophiolite (Colman et al., 2022). There are also cytochrome- dependent formate dehydrogenases (Sebban-Kreuzer et al., 1998). All formate dehydrogenases known so far, including HDCR, use Mo (in molybdopterin) or W (in tungstopterin) as a cofactor. The enzymatic mechanism of FDH is thought to involve binding of formate to Mo (or W) via its oxygen atoms (Hartmann et al., 2016), the abiotic mechanism is thought to involve bonding of the carbon atom in formate to Ni or Fe (Preiner et al., 2020).

The use of formate as a carbon source is a common theme in the effluent of serpentinizing systems. Although organisms have not yet been cultivated from hydrothermal vents that exhibit growth on formate, acetogens (Moon et al., 2021) and methanogens (Ferry et al., 1974) are known that use formate as their sole carbon and electron source, and some methanogens use formate as their sole carbon source (Costa et al., 2010; Lie et al., 2012). All indications are that formate based primary production is taking place at the submarine system of Lost City (Lang et al., 2018) and in some terrestrial serpentinizing systems including the Oman ophiolite (Colman et al., 2022), the Cedars (Suzuki et al., 2018), at Hakuba Happo (Nobu et al., 2023) and in the Prony Bay system, a freshwater fed submarine serpentinizing system that vents in seawater at a depth of about 50 m (Frouin et al., 2022), because there is no other carbon source reported to fuel primary production in these systems. For acetogens and methanogens living in these systems, formate is apparently the source of CO_2_ and a source of electrons for primary production via the acetyl-CoA pathway, which generates reduced carbon to support the growth of fermenters that live from microbial cell mass (Schönheit et al., 2016) and other heterotrophs in the system.

The results of microbial sampling studies from different sites are not simple to compare directly because community composition within sites can vary, and at very small scales, depending on the specific position of the sample within the vent, effluent seawater mixing in the case of submarine vents, presence or absence of O_2_ in the specific sample, the methods of analysis and other factors. For example, in the hyper-alkaline Strytan site, which has alkaline effluent but is not serpentinizing, anaerobes and the Wood Ljungdahl pathway of CO_2_ fixation dominate in inner regions of vents while O_2_-tolerant pathways dominate at the vent surfaces in contact with sea water (Twing et al., 2022). In the basement of Lost City alone, a broad diversity of phylotypes across sites sampled is observed (Brazelton et al., 2022). Methanogens are common in serpentinizing systems and can access carbon as formate (Fones et al., 2021). Quéméneur et al. (2023) showed that the abundance of certain prokaryotes is positively correlated to the H_2_/CH_4_ ratio in hyperalkaline springs and that bacteria that use H_2_ as their main energy source are the most abundant among the microbial communities of Prony Bay.

A recent study by Frouin et al. (2022) stands out among surveys of microbial communities from different serpentinizing systems because the authors aimed to identify community and physiological properties that i) are shared among different serpentinizing systems and that ii) distinguish serpentinizing systems from hydrothermal vents in non-serpentinizing systems. They found that serpentinizing systems tend to be colonized by diverse communities, and that bacterial groups encountered often include acetogens (Firmicutes) and methanogens like Methanosarcinales (Archaea), as originally discovered at Lost City. Frouin et al. (2022) found that Prony Bay was dominated by the acetogen *Bipolaricaulota*, which was also the main inhabitant of the Oman ophiolite community recently characterized by Colman et al. (2022) on the other side of the globe. Abundance of Firmicutes, which includes many acetogens, has been shown to be proportional to dissolved H_2_, however why that is still has to be determined (Quéméneur et al., 2023).

A common physiological theme in serpentinizing systems is the ubiquitous presence of enzymes for the acetyl-CoA pathway (or Wood-Ljungdahl pathway) of CO_2_ fixation. The Wood-Ljungdahl pathway is considered to be the most ancient pathway of carbon fixation (Fuchs and Stupperich, 1985; Fuchs and Stupperich, 1986; Berg, 2011; Fuchs, 2011). It is still the only exergonic pathway of CO_2_ fixation known. The reaction to the level of the energy-rich thioester


$${2CO}_{2}+8\left[H\right]+\text{CoASH}\to {CH}_{3}\text{COSCoA}+3H_{2}O$$

is exergonic with Δ*G*_o_*′* = –59.2 kJ/mol if 2[H] = H_2_ and slightly endergonic with Δ*G*_o_*′* = +13.2 kJ/mol if 2[H] = NADH (Fuchs, 1994). The NADH forming reaction is slightly exergonic in the direction of CO_2_, which is one reason that the pathway is so versatile among microbes (Fuchs, 1994; Zinder, 1994). It is the only pathway of CO_2_ fixation that does not require net ATP input (Fuchs, 2011) and that occurs in both bacteria and archaea (Berg et al., 2010). It is a joined pathway of carbon and energy metabolism in which acetogens and methanogens obtain their ATP from ion gradients that they generate in the process of the exergonic reduction of CO_2_ with electrons from H_2_ at MtrA-H in methanogens (Thauer et al., 2008) and at Rnf in acetogens that lack cytochromes (Müller et al., 2018). In addition, acetogens and some methanogens can generate ATP in the acetyl-CoA pathway using substrate-level phosphorylation via acetyl-phosphate (Schöne et al., 2022). This kind of combined carbon and energy metabolism is so far unique to acetogens and methanogens. The acetyl-CoA pathway can be a dedicated pathway of CO_2_ fixation (carbon metabolism) with no role in energy metabolism (ATP synthesis), for example in sulfate reducers that grow autotrophically, or in bacteria with phosphite-dependent energy metabolism (Mao et al., 2021). The acetyl-CoA pathway is a biochemical fossil that links acetogens and methanogens via functional, ecological and ancient evolutionary aspects (Martin, 2020; Schöne et al., 2022).

### 4.2. Electrons

**Figure 2:**
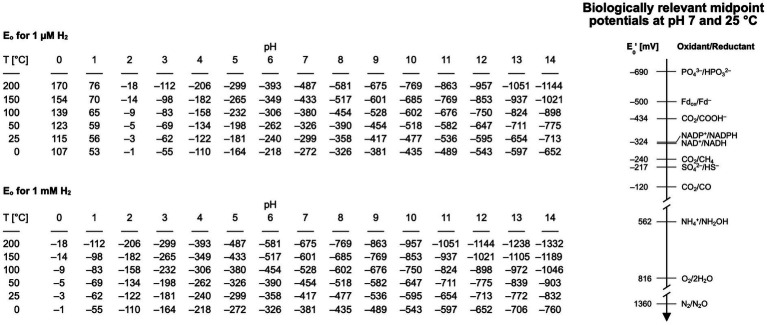
Range of redox potentials for two different concentrations of hydrogen (1 μM and 1 mM) over different pH ranges and temperatures. The pH has the biggest influence on the redox potential, followed by temperature and then hydrogen. Biologically relevant midpoint potentials are shown on the left, all of them measured at pH 7 (Vanysek, 2000; Schink et al., 2002; Lundblad and Macdonald, 2018; Martin et al., 2018; Madigan et al., 2021; Nelson and Cox, 2021).

The diffusible carrier electrons for reduction reactions in serpentinizing systems, H_2_, is present in overabundance. At Lost City the effluent H_2_ concentration is on the order of 10 mmol/kg [0.5 to 14 mmol/kg: (Proskurowski et al., 2008)], which is about 5–6 orders of magnitude more H_2_ than is required to support methanogenesis (Thauer et al., 2008). Because of the high alkalinity and high H_2_ concentration, the calculated redox potential of serpentinizing effluent can be in the range of –800 mV (Boyd et al., 2020), sufficient for most biologically relevant reduction as shown in Figure 2, with the exception of the phosphate/phosphite couple, where the midpoint potentials become more negative with increasing pH (Bernhard Schink, pers. comm.). Alkalinity favors the dissociation of H_2_ into protons and electrons by consuming protons from the reaction H_2_ → 2e^–^ + 2H^+^. Electrons from H_2_ enter metabolism via hydrogenase. All hydrogenases except the Fe hydrogenase of methanogens (Thauer et al., 2008; Shima et al., 2011), reduce 4Fe4S clusters and most of them generate reduced ferredoxin as the reaction product. The redox potential needed to reduce ferredoxin as the source of electrons in metabolism is on the order of –500 mV. Suzuki et al. (2018) found no hydrogenases in metagenomes from The Cedars, raising the possibility that other routes of ferredoxin reduction might be possible under such reducing conditions. Studies of microbes that grow on native iron and on electrodes suggest that side activities of extracellular enzymes might be able to substitute for hydrogenases (Deutzmann et al., 2015). Early studies by Menon and Ragsdale (1996) indicated that acetyl-CoA synthase and pyruvate synthase have latent hydrogenase side activity, and the H_2_-producing side reaction of nitrogenase (Hu and Ribbe, 2016) is well known. All of those side activity hydrogenases have FeS or NiFeS clusters that are likely candidates for the moonlighting active site. Metals (Fe, Ni, Co) and metal oxides can serve as hydrogenases, activating H_2_ by splitting it into two metal bound hydrogen atoms for CO_2_ reduction (Preiner et al., 2020; Belthle et al., 2022; Beyazay et al., 2023a,b) or for NAD^+^ reduction (Henriques Pereira et al., 2022).

**Figure 3:**
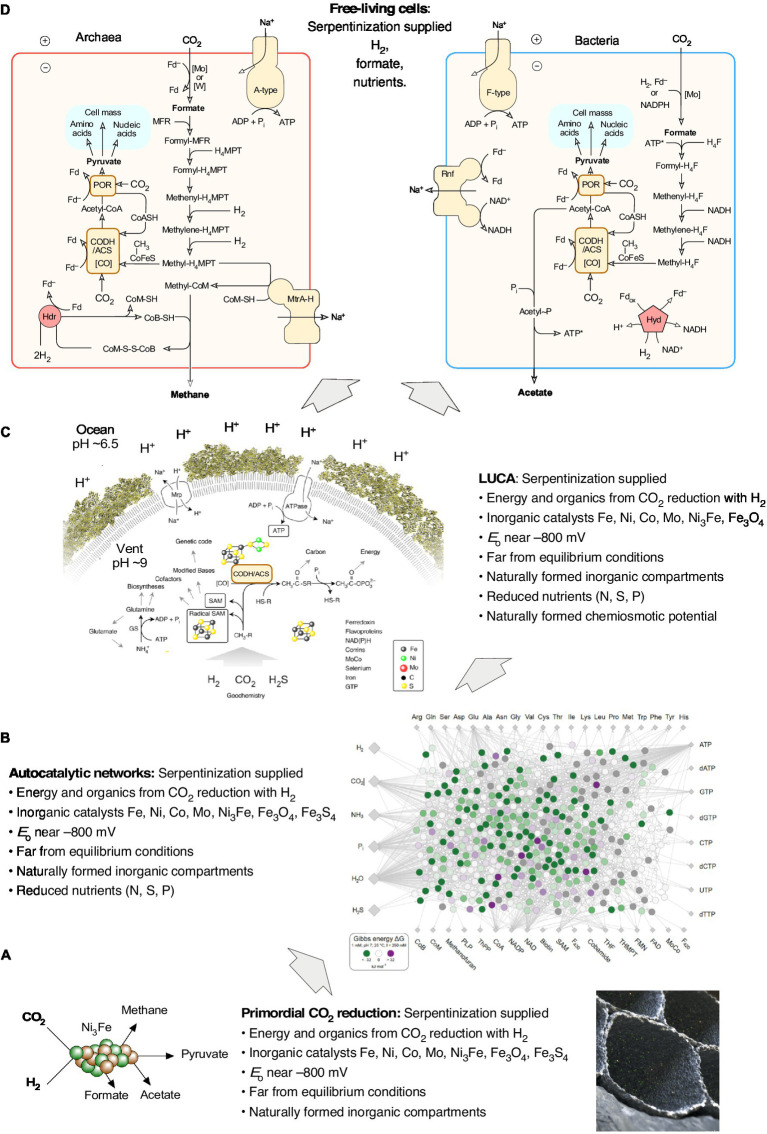
Contributions of serpentinization to the origin of free-living cells. **(A)** The H_2_ produced in serpentinization synthesizes awaruite, Ni_3_Fe (Preiner et al., 2018). Awaruite is a highly efficient catalyst for the conversion of H_2_ and CO_2_ into formate, acetate and pyruvate — the backbone of microbial metabolism in anaerobic autotrophs that obtain their carbon and energy via the acetyl-CoA pathway — the reaction takes place overnight in water at pH 8-10 and 70-100°C in the dark using aqueous H_2_ concentrations of 5 mM, similar to 10 millimolar H_2_ observed at Lost City (Preiner et al., 2020). At that H_2_ concentration and pH, the midpoint potential of the H_2_/H^+^ couple is on the order of –600 mV (see Figure 2), in Lost City the redox potential has been calculated to be in the order of –800 mV (Boyd et al., 2020), sufficient to reduce both CO_2_ to formate (E_o_’ = –450 mV) and to reduce ferredoxin (E_o_ = *ca.* –500 mV in cells). These low values of E_o_ are the reason why electron bifurcation is not required for CO_2_ reduction with H_2_ (E_o_’ = –414 mV) in the reaction shown in the panel (Martin, 2022), but is strictly required in enzyme based reduction in cells (Buckel and Thauer, 2018; Müller et al., 2018). Naturally formed inorganic microcompartments (an artist’s impression is shown) can concentrate the organic products around their site of synthesis. **(B)** Autocatalytic networks are objects of molecular (self) organization. Because of the specificity and efficiency observed for their formation from H_2_ and CO_2_ using Ni_3_Fe or Fe_3_O_4_, formate, acetate and pyruvate serve as elements of the food set (F) in food- (F-) generated reflexively autocatalytic networks, abbreviated as RAFs. RAFs can be identified in the metabolism of modern cells (Sousa et al., 2015; Xavier and Kauffman, 2022) and in acetogens and methanogens, cells that obtain their carbon and energy from H_2_ dependent CO_2_ reduction via the acetyl CoA pathway (Xavier et al., 2021). The reactions that are required to synthesize 20 amino acids, the (unmodified) bases of DNA and RNA, and 18 cofactors are called the autotrophic core (Wimmer et al., 2021a). Of the 400 reactions in the autotrophic core, 97 % reactions are exergonic under the reducing and far-from-equilibrium conditions of serpentinizing hydrothermal vents, as indicated by green dots on reactions of the network [image from Wimmer et al. (2021b)]. Serpentinization also provided reduced N, S and possibly P species as nutrients (see corresponding sections in the text). Natural inorganic compartments could have promoted the formation of autocatalytic networks by generating high reactant and product concentrations from specific food sets (Martin and Russell, 2007). Autocatalytic networks help to bridge the gap between reactions catalyzed by simple inorganic compounds and reactions catalyzed by peptides synthesized on ribosomes. **(C)** The last universal common ancestor, LUCA, possessed the universal genetic code and ribosomes was able to synthesize proteins as catalysts. Genomic reconstructions of LUCA (Weiss et al., 2016, 2018) indicate that the energy required for the synthesis of encoded peptide catalysts (protein synthesis cost 4 ATP per peptide bond) stemmed from substrate level phosphorylation, SLP (Weiss et al., 2016). Serpentinization provided H_2_ for CO_2_ reduction. The thermodynamics of laboratory scale bifurcation-independent acetate synthesis from H_2_ and CO_2_ are sufficient to enable SLP (Preiner et al., 2020). Serpentinization generates naturally occurring pH gradients between the alkaline effluent and the nearly neutral ocean water (see main text), the magnitude and polarity of the gradients are sufficient to drive an ATP synthase. The rotor stator ATP synthase is as universal among cells as the ribosome (Martin and Russell, 2003), it was present in LUCA (Weiss et al., 2016) and, inserted into a hydrophobic layer would provide the cytosol with a very high energy charge to support for protein synthesis. The origin of the genetic code is handled here as a given, not as an explanandum, it is not evident how serpentinization (or any other geochemical process) would specifically foster the origin of the genetic code, whose structure is determined by aminoacyl-tRNA synthetases (Carter and Wolfenden, 2015), bases in tRNA (Schimmel et al., 1993) and the peptidyl transferase site of the protoribosome (Bose et al., 2022). **(D)** By coupling H_2_ dependent CO_2_ reduction to the formation of ion gradients (pumping), and with the integration of flavin based electron bifurcation into the reduction of ferredoxin by H_2_ in a stoichiometrically balanced energy metabolic reaction, the ancestors of acetogens (LBCA) and methanogens (LACA) could emerge as free-living cells. The lipids of bacteria and archaea are different in chemistry and biosynthesis, they evolved independently from LUCA en route to LACA and LBCA. See also Martin (2012, 2020) and Martin and Sousa (2016). Note the position of H_2_, CO_2_, formate, acetate, pyruvate and methane in panel A and panel D. In free living cells, various properties provided in different phases of biochemical evolution by serpentinization are replaced by biogenic molecules and catalysts. In all phases of the figure, the energy is supplied by serpentinization. In panel **(D)** the archaeal physiological map is for Methanothermobacter marburgensis, from Thauer et al. (2008), the bacterial map is for Acetobacterium woodii, from Schuchmann and Müller (2014).

The highly reducing conditions of serpentinizing hydrothermal vents are key to origins because CO_2_ has to be converted to organic carbon. There are, however, also alternative views about the source of energy at origins in serpentinizing hydrothermal systems. Duval et al. (2021), for example, argue that the energy required for the origin of metabolism was not exergonic H_2_-dependent CO_2_ reduction catalyzed by reduced transition metals as it occurs in the laboratory (Weiss et al., 2016; Preiner et al., 2020; Wimmer et al., 2021b; Beyazay et al., 2023a) and in acetogen and methanogen metabolism, but that instead the energy at origins stemmed from nitrate-dependent methane oxidation with the mixed valence iron oxide fougerite (green rust), acting as the catalyst (Duval et al., 2021) in alkaline hydrothermal systems. How energy release from methane oxidation is coupled to either prebiotic organic synthesis or carbon metabolism is not explained by Duval et al. (2021), changes in Gibbs free energy for the fougerite dependent reactions have not been presented, and abiotic laboratory versions of the fougerite dependent carbon assimilation reactions have not been reported. In laboratory versions of the acetyl-CoA pathway (Preiner et al., 2020; Beyazay et al., 2023a) and in acetogens and methanogens *in vivo* (Figure 3), energy for ATP synthesis is released in the exergonic H_2_ dependent synthesis of acetate, pyruvate and methane from CO_2_.

### 4.3. Nitrogen and sulfur

Frouin et al. (2022) looked carefully at sources and pathways for nitrogen but found no consistent trend. The source of nitrogen is still not well resolved in serpentinizing systems and might differ across systems. In very recent work, Shang et al. (2023a,b) found that experimental serpentinization reactions in the laboratory readily reduce N_2_ to NH_3_ in very large amounts and on time scales approaching 30 days. Cells are about 50% carbon and about 10% nitrogen by dry weight (Heldal et al., 1985), hence there has to be access to a nitrogen supply for primary production in serpentinizing systems. The simplest solution is that N_2_ present in the water circulating through serpentinizing systems is reduced by nitrogenase. Most studies have detected nitrogenase genes among the microbiota of serpentinizing systems, though nitrate reduction can also be detected (Frouin et al., 2022). Trutschel et al. (2022) reported high concentrations of ammonia at Ney Springs. Ammonia has also been reported in the Oman ophiolite system (Rempfert et al., 2017), but in both cases the source of the ammonia is not resolved, with decaying organic matter within the system being the most widely discussed source. With the new report by Shang et al. (2023a), abiotic sources of NH_3_ need to be considered in natural systems (Shang et al., 2023a).

The state of nitrogen in the primordial atmosphere was N_2_ (Sossi et al., 2020). In an origins context, before the existence of biological N_2_ fixation via nitrogenase, the source of nitrogen would have to be geochemical. Three sources are discussed: High pressure high temperature N_2_ reduction by deep geochemical Haber-Bosch like processes (Brandes et al., 1998), low pressure low temperature FeS-mediated N_2_ reduction processes (Dörr et al., 2003) and serpentinization-related geochemical processes. Ménez et al. (2018) reported the presence of abiotic tryptophan and other amino acids in the Atlantis massif that hosts the Lost City hydrothermal field. The exact route of synthesis is not yet known but might involve iron-dependent Friedel-Crafts like syntheses. The significance of amino acid synthesis in the crust for theories that life arose at hydrothermal vents is evident (Baross, 2018).

Lost City does not appear to emit abiotic amino acids in its effluent, but the terrestrial hydrothermal system Hakuba Happo does. Nobu et al. (2023) recently reported the presence of glycine (5 nM) in the effluent of Hakuba Happo which is likely of abiotic origin. Notably, there were no other amino acids in the effluent. Were glycine of biotic origin, the 19 other amino acids should be present. Some of the microbes that Nobu et al. characterized from Hakuba Happo, *ca.* Lithacetigenota, possessed genes for glycine reductase, an unusual selenoenzyme that synthesizes acetyl phosphate from glycine and P_i_ (Andreesen, 2004). The acetyl phosphate can be used for acetyl-CoA (carbon metabolism) or ATP synthesis via substrate level phosphorylation. Although the concentration of glycine in the Hakuba Happo effluent was low, it was sufficient to render the glycine reductase pathway thermodynamically favorable under the conditions of the vent. No archaea were detected in Hakuba Happo, but glycine reductase utilizing Lithacetigenota were detected at the Cedars (Nobu et al., 2023).

The glycine reductase variant of carbon assimilation and energy metabolism employed by Lithacetigenota starts with environmental glycine. It is distinct from the H_2_-dependent glycine reductase pathway of autotrophic CO_2_ fixation used by *Desulfovibrio desulfuricans* (Sánchez-Andrea et al., 2020). The findings of Ménez et al. (2018) indicate that geochemical nitrogen fixation to amino acids is possible. The findings of Nobu et al. (2023) suggest that it is ongoing today, continuously in the hyperalkaline serpentinizing environment of Hakuba Happo.

Sulfur is a trace element in cells. It is highly volatile in various oxidation states and readily eluted from rocks as H_2_S or HS^–^ during serpentinization, but sulfate is also a common constituent of effluent and sulfate reducers are very common in serpentinizing systems (Reveillaud et al., 2016; Lang et al., 2018). Sulfur is an ancient substrate of energy metabolism (Rabus et al., 2006; Liu et al., 2012) and fulfills important catalytic functions in many cofactors (Kirschning, 2021). It is a component of ancient metabolism. Note however that the synthesis of formate, acetate and pyruvate from H_2_ and CO_2_ under simulated hydrothermal conditions using Ni_3_Fe or Fe_3_O_4_ as catalysts requires no participation of sulfur (Preiner et al., 2020; Belthle et al., 2022; Beyazay et al., 2023a,b).

Today, nitrogen and sulfur compounds that occur in hydrothermal systems serve as terminal electron acceptors in anaerobic respiratory chains (Calisto and Pereira, 2021). At the origin of metabolism, CO_2_ was arguably the most important electron acceptor because the synthesis of organic compounds was required in order to get metabolic pathways established. This underscores the evolutionary significance of the acetyl CoA pathway in acetogens and methanogens: it generates the starting compounds of metabolism while releasing energy that can be harnessed to synthesize ATP. In that sense, acetogenesis and methanogenesis are cytochrome-free and quinone-free anaerobic respirations that use CO_2_ as the terminal acceptor in a reaction sequence that also generates a net gain of acetyl CoA. Anaerobic respirations that reduce sulfur and nitrogen compounds require siroheme, cytochromes and quinones, acetogenesis and methanogenesis do not, they are simpler in that regard. Coupled carbon and energy metabolism in the acetyl-CoA pathway using CO_2_ as the electron acceptor probably preceded a differentiation into dedicated carbon metabolism and dedicated energy metabolism involving cytochrome dependent respiratory chains that use nitrogen and sulfur compounds as terminal acceptors.

### 4.4. Phosphorus: phosphate and phosphite

Phosphate has always presented problems in prebiotic chemistry because of its low solubility in contact with calcium to generate apatite, which is more or less insoluble, leading to various suggestions, that certain kinds of carbonate rich lakes existed on the primordial Earth that maintained phosphate in solution (Toner and Catling, 2020). The recent findings of Frouin et al. (2022) from Prony Bay place the phosphate problem in a fundamentally different light. They estimate that about half of the microbes that they sampled from Prony Bay possess the genes required for the metabolism of phosphonates and they found that phosphonate metabolism was fairly common in microbial communities from serpentinizing systems. What are phosphonates? Phosphonates are compounds that contain a C–P bond and are so far only known to be synthesized by microbes via a pathway that starts from phosphoenolpyruvate. Phosphonates can have concentrations in open ocean water that account for up to 25% of dissolved organic phosphorus (Acker et al., 2022). Their exact function is still not known, but they might represent a means of sequestering phosphorus among community members (Metcalf et al., 2012). That proposal has however recently been challenged because the phosphonate synthesis and utilization genes are so common: phosphonates might also mediate biological interactions at the cell surface (Acker et al., 2022). The question posed by Frouin et al. (2022) is: What are microbes from serpentinizing systems doing with a high frequency of phosphonate metabolizing enzymes?

Moreover, in some of the Prony Bay metagenomes, the gene for an additional enzyme, NAD^+^-dependent phosphite oxidoreductase, *ptxD*, was often inserted into the phosphonate metabolizing operon (Frouin et al., 2022). The *ptxD* gene product converts phosphite and NAD^+^ to phosphate and NADH (Vrtis et al., 2001). Frouin et al. (2022) noted the ability of Prony Bay microbes to utilize reduced phosphorous compounds. They were mainly concerned with the question of what the source of phosphonates in the Prony Bay fluid might be. While about half of the genomes from the Prony Bay vents contained genes related to phosphonate metabolism, metabolizing operons represent a frequency that is significantly higher than average for marine microbes (Frouin et al., 2022), it is not orders of magnitude higher: up to 30 % of marine microbial genomes have the potential for phosphonate degradation (Acker et al., 2022).

New findings by Pasek et al. (2022) suggest that the phosphite oxidase gene, *ptxD*, that Frouin et al. find inserted into the phosphonate (phn) operon might hold an important clue. Why is phosphite relevant? Two reasons: First, phosphite is much more soluble than phosphate. One solution to the “phosphate problem” of early evolution is that phosphorus in the ancient oceans was present in the form of the much more soluble phosphite ion (Schwartz, 2006). Studies by Pasek and colleagues have suggested that phosphite might have been more prevalent on the early Earth than it is today. Archaean oceans may have contained substantial amounts of phosphite in addition to phosphate (Pasek et al., 2013; Herschy et al., 2018). If phosphite was present in early environments but phosphate was needed, enzymatic phosphite oxidation would have been needed. Might the presence of phosphonates in the ocean, with P^+3^ oxidation state (like phosphite) and oxidation to phosphate (P^+5^) in the cytosol, reflect a relic of ancestral marine environments? It is possible.

Second, where does phosphite in the environment come from such that so many microbes should have genes for phosphite metabolism (Schink et al., 2002)? In older papers, a source of ancient phosphite was originally suggested to be schreibersite from meteorite bombardments (Pasek et al., 2013). Schreibersite is a highly reactive phosphide mineral (Ni, Fe)_3_P, that is indeed only known from meteorites (Pasek, 2008). But phosphite oxidation genes are commonplace today (Schink and Friedrich, 2000; Vrtis et al., 2001), being found in roughly 1.5 % of all genomes (Ewens et al., 2021), whereas huge schreibersite spreading impacts are rare at best. This clearly indicates that there are sources of phosphite in the environment that do not require bolide impacts.

The midpoint potential for the reduction of phosphate to phosphite is –690 mV at pH 7 and 25°C (Schink et al., 2002) (see Figure 2) but becomes more negative with higher pH (Bernhard Schink pers. comm.). Considering the measured and calculated reduction and midpoint potentials for serpentinizing systems is within the range of –435 to –830 mV, the reduction of phosphate to phosphite might not be within the range covered by H_2_ in modern serpentinizing systems, […] but might lie within the range of serpentinization processes that generate H_2_ on early Earth. This brings us to a question relevant for modern microbial communities and for life’s origins: do serpentinizing systems actually generate phosphite? The answer appears to be yes (Pasek et al., 2022), although phosphite has not been reported to be present directly in the effluent of serpentinizing systems. Pasek et al. (2022) measured phosphorus species in samples of rock from formations altered by serpentinization, they found that 20–50 % of the total P in some samples was phosphite (the remainder was phosphate). That corresponds to a substantial amount of phosphite stemming from serpentinization. The implications of i) genes for phosphite oxidation being present in microbes from serpentinizing systems (Frouin et al., 2022) and ii) phosphite being a component of rock in serpentinizing hydrothermal systems (Pasek et al., 2022) suggest that there is phosphite available in serpentinizing systems and that microbes that live there are using it. Could they be using this as a soluble source of P for enzymatic phosphate synthesis? If so, this would tie together some loose ends in phosphate metabolism and early evolution.

It is possible that genes for phosphite uptake and conversion into its biologically more useful form, phosphate, might reflect an ancient, possibly even ancestral state of phosphorus metabolism. As Buckel (2001) put it: “*At the time of the origin of life, about 3.8 billion years ago, phosphites could have been more important than today*.” This would mean that the highly exergonic conversion of phosphite into its biologically relevant form, phosphate, occurred inside cells. But in early chemical evolution, before there were enzymes, was there a role for phosphite? This has not yet been extensively studied. Incorporation of phosphate into the fabric of early protobiochemical synthesis still presents a few sticking points in early metabolic evolution (Martin, 2020), environmental phosphite offers some alternative entry routes (Buckel, 2001).

Finally, the relevance of phosphite is underscored by the circumstance that it is a growth substrate for microbes, specifically for anaerobic autotrophs (Schink and Friedrich, 2000). Schink et al. (2002) characterized *Desulfotignum phosphitoxidans*, that grew autotrophically with phosphite as the sole electron donor and CO_2_ as the sole electron acceptor. The energy-yielding metabolic reaction was the synthesis of phosphate with some acetate being produced as well. *D. phosphitoxidans* can also grow as a sulfate reducer that uses the acetyl-CoA pathway. In the absence of sulfate, its metabolism was that of an acetogen with energy generated by CO_2_ reduction to acetate but using phosphite instead of H_2_ as a source of electrons (Schink et al., 2002). Another phosphite oxidizer was recently isolated, but it was an autotrophic phosphite specialist — the strict anaerobe grew as a phosphite-dependent autotroph, using only phosphite as the electron donor and only CO_2_ as the electron acceptor (Mao et al., 2021).

Buckel (2001) suggested that the highly exergonic process of phosphite oxidation might be coupled to substrate level phosphorylation, which is thermodynamically possible, but has not yet been shown so far. Phosphite in the environment appears to be derived from serpentinization. It is the only reductant other than H_2_ that is known to fuel chemolithoautotrophic growth using the acetyl-CoA pathway. This links phosphite to serpentinization and ancient acetogenic physiology. Phosphite has a sufficiently negative midpoint potential that electron bifurcation would not be needed to reduce ferredoxin in cells that use phosphite, but its enzymatic oxidation product is NADH, which would require electron bifurcation for ferredoxin reduction.

## 5. Serpentinization, biochemical networks, physiology, and autotrophic origins

Under theories for autotrophic origins, the first free-living cells were able to synthesize all of their components from CO_2_, a reductant (H_2_), a nitrogen source and inorganic salts. It is a long way from the H_2_ + CO_2_-dependent synthesis of formate, acetate and pyruvate to the synthesis of free-living autotrophic cells, which require on the order of 1,500 genes and proteins to survive. Chemical reaction systems called autocatalytic networks are typically seen as intermediates in that evolutionary transition. Autocatalytic networks are objects of molecular (self-)organization. They involve catalytic properties of compounds within the network that act as simple catalysts to accelerate reactions within the network such that more products within the network, hence more catalysts, arise from the starting compounds (typically called the food set for the network) (Hordijk et al., 2011). Autocatalytic sets called RAFs (reflexively autocatalytic food-generated networks) are of particular interest for origins because they can be easily modeled on the computer (Hordijk and Steel, 2017) and because they can be detected in the metabolic maps of modern microbes (Sousa et al., 2015). Xavier et al. (2020) detected RAFs in the metabolic maps of a well-curated acetogen and a well-curated methanogen. The sizes of those networks were 394 reactions (acetogen) and 209 reactions (methanogen) respectively. The acetogen and methanogen RAFs overlapped by 172 reactions that correspond to the RAF of their last common ancestor, which is effectively the RAF of the last universal common ancestor LUCA, which was highly enriched for transition metal catalysts and carbon metal bonds.

Note that the structure of RAFs (mathematical constructs) and the structure of real metabolic maps are not identical, such that a number of simplifying assumptions have to be made in order to apply RAF-detecting algorithms to real metabolic maps; Sousa et al. (2015) spelled out 11 simplifying assumptions that need to be taken into account when identifying RAFs in metabolic maps. The RAF of LUCA had the interesting property that RNA nucleobases arise from metabolic networks, but metabolic networks do not arise from bases, in line with a metabolism first view of origins as opposed to an RNA first view (Xavier et al., 2020). Xavier and Kauffman (2022) recently found that small-molecule autocatalytic networks are present in over 6,000 metabolic maps investigated, suggesting that autocatalytic networks are not only ancient, tracing to LUCA, but that they are also universal in metabolism.

The requirement for catalysis in RAFs means that cofactors are highly represented in RAFs detected computationally and that essential products that are required for life might not be included in a RAF. For example, the RAF of LUCA (Xavier et al., 2020) did not generate all amino acids or cofactors. How many reactions did primordial metabolism encompass? Autotrophs would need to synthesize all small molecules of metabolism themselves. Wimmer et al. (2021a) found that only 404 reactions are required to synthesize the 20 canonical amino acids, the bases of RNA and DNA (excluding modifications) and the 18 cofactors of ancient metabolism from H_2_, CO_2_, NH_3_, H_2_S, H_2_O and P_i_. Furthermore, 97 % of those reactions were exergonic under the conditions of a serpentinizing hydrothermal vent (alkaline, high pH, H_2_ as a reductant), indicating that there is a natural tendency for reaction of metabolism to unfold under far from equilibrium hydrothermal conditions (Wimmer et al., 2021b). A theoretical study by Nunes Palmeira et al. (2022) involving autocatalysis in a simplified metabolic model concluded that various lines of evidence indicate that metabolism emerged from a geochemical protometabolism fueled by H_2_ and CO_2_.

Reconstructions of metabolism are also in line with an autotrophic origin of life at hydrothermal vents. Mei et al. (2023) and Williams et al. (2017) found that the ancestral physiology of archaea was likely hydrogen-dependent methanogenesis. Xavier et al. (2021) found that the ancestral physiology of bacteria was likely hydrogen-dependent acetogenesis. Coleman et al. (2021) found that the acetyl-CoA pathway reconstructs to the root of bacterial phylogeny although there is some discussion about the method they employed to infer the position of the root in the bacterial tree (Bremer et al., 2022). Schöne et al. (2022) found that with considerable genetic manipulation, a methanogen could be converted into acetogenic physiology, reflecting ancient physiological connections between the two groups and possibly uncovering an ancestral state of ATP synthesis via substrate level phosphorylation prior to the origin of chemiosmotic ATP synthesis. These findings that trace the exergonic reactions of H_2_ with CO_2_ in acetogenesis and methanogenesis to the first cells are consistent with genomic reconstructions of LUCA indicating that LUCA arose from similar reactions at a serpentinizing hydrothermal vent and that the most ancient lineages of anaerobes are acetogens and methanogens (Weiss et al., 2016). While there are numerous genomic reconstructions of LUCA present in the literature, they almost all focus on attributes and processes that are universal or nearly so among cells such as protein synthesis, ribosomal proteins, conserved pathways, and nucleic acids (Goldman et al., 2013) or genetics and lateral gene transfer (Crapitto et al., 2022). The studies of Weiss et al. (2016, 2018) took a different approach by looking carefully at physiology rather than universal gene distribution, in order to extract information about the environment in which LUCA arose and diversified and the presence of ancient physiological attributes such as transition metal catalysis, autotrophy, exergonic H_2_-dependent CO_2_ reduction, geochemical methyl groups, substrate level phosphorylation, and chemiosmotic ATP synthesis utilizing geochemical ion gradients. The studies of Weiss et al. (2016, 2018) are sometimes criticized for inferences about thermophily and use of the term “progenote “(Gogarten and Deamer, 2016), but no other study of LUCA makes a statement on the origin of ATP to drive any of the processes that are ascribed to LUCA by gene and genome-based inference. To drive ATP synthesis, exergonic chemical reactions of compounds in the environment have to be harnessed and the energy conserved as a biologically useful form such as thioesters, acyl anilides, acyl phosphates or ATP. In our model for LUCA and origins, LUCA’s energy conservation and ATP synthesis comes from serpentinization.

### 5.1. Serpentinization: brimming with the energy of life

There are only two basic ways that cells conserve energy as high energy phosphate bonds: substrate level phosphorylation (Decker et al., 1970) and chemiosmotic energy harnessing using a rotor-stator ATP synthase (Walker et al., 1982; Walker, 2013). Serpentinizing systems provide a chemical environment that can support the origin of both forms of biological energy conservation.

Substrate level phosphorylation usually involves the generation of an acyl phosphate (or enol phosphate) bond during the oxidative breakdown of reduced carbon compounds. The classical example is the synthesis of the acyl phosphate bond in 1,3-bisphosphoglycerate at the reaction catalyzed by glyceraldehyde-3-phosphate dehydrogenase by oxidizing the aldehyde group on C1 of glyceraldehyde-3-phosphate with NAD^+^ to form a thioester bond between the enzyme and the substrate, which is cleaved via phosphorolysis, generating the acyl phosphate bond at C1 which can phosphorylate ADP to generate ATP. There are also reductive routes of SLP, for example the conversion of the thioester bond in acetyl-CoA from the (reductive) acetyl-CoA pathway to acetyl phosphate, which can also phosphorylate ADP. Although the direct synthesis of acyl phosphates from thioesters has so far not been reported, it has long been known that acyl phosphates can phosphorylate ADP without enzymes (Kitani et al., 1991). More recently, Whicher et al. (2018) have shown that thioacetate can readily react with P_i_ to form acyl phosphates without enzymes, although thioacids are not known in central carbon or energy metabolism. The synthesis of acetate and pyruvate from H_2_ and CO_2_ under serpentinizing vent conditions using catalysts synthesized at hydrothermal vents — awaruite and magnetite — is facile whereby there is furthermore enough energy released in the synthesis of acetate to energetically support SLP (Preiner et al., 2020; Belthle et al., 2022; Beyazay et al., 2023a,b). Reductive SLP also takes place via acetyl phosphate synthesis at the glycine reductase reaction (Andreesen, 2004) and the subsequent phosphorylation of ADP. Otherwise, net ATP synthesis involves either SLP via oxidation reactions of organic compounds (Müller, 2003; Martin and Thauer, 2017), which are thermodynamically unfavorable under the reducing conditions of serpentinizing systems, or it involves chemiosmotic ATP synthesis using the rotor-stator ATP synthase.

The rotor-stator ATP synthase is universal among cells. It generates ATP by the passage of protons (or Na^+^) from the outside of the cell through the ATP synthase to the inside of the cell. The passage of protons through the ion channel of the stator subunit *a* and the subsequent protonation and deprotonation of an acidic residue in each of the c subunits of the ring-shaped rotor sets the rotor of the ATP synthase in rotation, inducing conformational changes in the catalytic subunits of the “head,” changes that forge a phosphoanhydride bond between ADP and P_i_ and lead to release of ATP from the enzyme. Three ATP molecules are synthesized per complete 360° rotation of the head, this requires roughly 12 protons traversing the enzyme. This mechanism of ATP synthesis is universal in bacteria and archaea, it is furthermore reversible such that cells can expend ATP that they obtain from SLP to generate ion gradients for the import of small molecules (Franklin, 1986) or to generate reduced ferredoxin (Buckel and Thauer, 2013). The ion gradients that modern cells use to power chemiosmotic ATP synthesis are generated by membrane integral ion pumping proteins, or coupling sites, that pump protons (or Na^+^) from the inside of the cell to the outside. There are two ways that pumping can be achieved: i) with the help of conformational changes within membrane-integral domains, and ii) with the help of what Mitchell called vectorial chemistry (Mitchell, 1991). In the former, the general mechanism (conformational change) is the same as that for the ATP synthase, except that the energy for conformational change and pumping stems from exergonic redox reactions that are part of the overall bioenergetic reactions of the cell, for example methane synthesis from H_2_ and CO_2_ (in methanogens) or acetate synthesis from H_2_ and CO_2_ (in acetogens). The second, evolutionarily more advanced mechanism (vectorial chemistry) requires the existence of quinones or quinone analogs (Berry, 2002), membrane soluble redox factors that can accept an electron pair and two protons on the cytosolic side and transfer the electron pair to an acceptor on the outside of the cell, thereby transferring protons from the inside of the cell to the outside merely by the relative position of hydride donor and hydride acceptor across the membrane. Ancient lineages of methanogens and acetogens lack quinones. They generate their ion gradients solely via conformational changes in membrane proteins (pumping).

It is a very curious observation that the principle of harnessing ion gradients for ATP synthesis and the enzyme that does it, the rotor-stator ATP synthase, are as universally conserved across all bacteria and archaea as the ribosome and the genetic code itself. This indicates that ion gradient harnessing was present in LUCA. LUCA could *use* ion gradients. Yet there is no similar form of conservation to indicate that LUCA could *generate* ion gradients. But if LUCA arose and existed at a serpentinizing vent, as much other evidence summarized above indicates, there is also no need to assume that LUCA had to generate ion gradients by itself, because serpentinization generates geochemical ion gradients continuously and for free that remain stable for the duration of the serpentinization process. Recalling that Lost City has been actively serpentinizing for roughly 100,000 years (Ludwig et al., 2011; Denny et al., 2016), a stable geochemically generated pH gradient of the right polarity (alkaline, lower proton concentration on the inside) and sufficient magnitude (cells synthesize ATP with the help of a pH difference of about one pH unit) (Tran and Unden, 1998; Silverstein, 2014) would present a continuous environmental setting in which natural chemiosmotic potential existed and could be harnessed (Martin and Russell, 2007; Martin, 2012).

This natural chemiosmotic energy source would have allowed the ancestral ATP synthase to function but would have required the evolution of protein based pumping mechanisms in the ancestors of the archaea and the bacteria in order for LACA (Last Archaeal Common Ancestor), (a methanogen) and LBCA (Last Bacterial Common Ancestor) (an acetogen) to make the transition to the free-living state. Methanogens without cytochromes pump with energy derived from H_2_-dependent CO_2_ reduction. The pumping reaction takes place at the methyl transferase step catalyzed by the membrane protein MtrA-H, which in a cobalamine-dependent reaction transfers a methyl group from a nitrogen atom in methyl-H_4_MPT to a sulfur atom in coenzyme M; the free energy of the reaction Δ*G*_o_′ is –30 kJ·mol^–1^ and pumps two Na^+^ ions from the inside of the cell to the outside (Thauer et al., 2008). This energy conserving reaction is universal among H_2_-dependent methanogens and likely represents their ancestral state. In acetogens without cytochromes, the pumping reaction is energetically driven by H_2_-dependent CO_2_ reduction and is catalyzed by the membrane protein Rnf, which pumps Na^+^ from the inside of the cell to the outside while transferring electrons from reduced ferredoxin to NAD^+^ (Biegel et al., 2011) with a Δ*G*_o_′ of –34.7 kJ·mol^–1^ (Kuhns et al., 2020).

**Figure 4:**
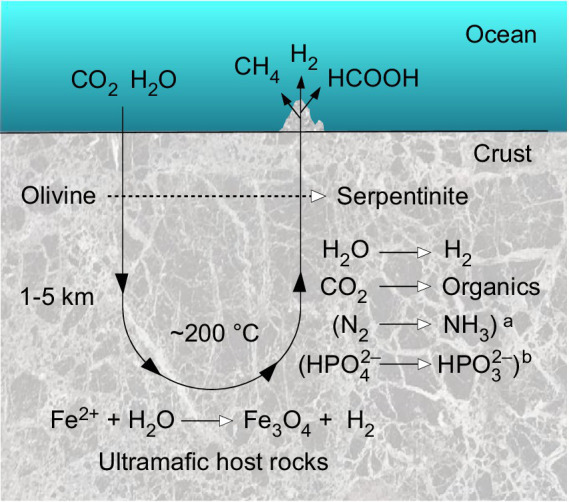
Serpentinization is a planetary scale reducing agent. The figure is modified from (Preiner et al., 2018). During serpentinization, surface water (seawater or freshwater) is drawn down into cracks in the crust where it reacts with ultramafic rocks at temperatures of about 200°C. During the reaction, Fe^2+^ in olivine is oxidized by water to Fe^3+^, generating iron oxides in serpentinite and magnetite, Fe_3_O_4_, as well as H_2_, diffusible reductant (Sleep et al., 2004; Bach et al., 2006; Lang et al., 2010). The reduction process converts water to H_2_, CO_2_ to formate and methane as well as other organics, and in newer studies of laboratory scale serpentinization, it converts N_2_ to NH_3_ (Shang et al., 2023b). Simulated geochemical systems using Co and Fe as catalysts can generate hydrocarbons from bicarbonate at high temperature and pressure (He et al., 2021). Although any kind of hydrophobic compounds could serve as insulators for the function of an ATP synthetase, long chain hydrocarbons are an option prior to the origin of genetically encoded enzymes for lipid synthesis. ^a^The reduction of N_2_ to NH_3_ is indicated in parentheses because this has not been directly shown in modern serpentinizing systems, […] but it occurs in laboratory scale serpentinization and very likely occurred in the Hadean crust […] (Shang et al., 2023a,b). ^b^Phosphate to phosphite is shown in parentheses because it is a recent discovery that serpentinzing systems might be able to generate it because naturally occurring serpentinized rocks can contain phosphite (Pasek et al., 2022); as with N_2_ reduction to NH_3_ the reaction has not been demonstrated in serpentinizing systems today.

By coupling H_2_-dependent CO_2_ reduction to ion gradient formation, cells were able to generate their own ion gradients, were no longer dependent on geochemical ion gradients for ATP supply and could make the transition to the free-living state. The machinery to generate ion gradients by coupling exergonic reactions of H_2_-dependent CO_2_ reduction to ion pumping arose independently in the lineages leading to LBCA and LACA, because different steps were harnessed and unrelated enzymes were used. Note that both acetogens and methanogens require flavin based electron bifurcation to generate reduced ferredoxin for CO_2_ reduction (Buckel and Thauer, 2018; Müller et al., 2018), but this is not required in CO_2_ reduction promoted by metal catalysts (e.g., Ni_3_Fe, Fe_3_O_4_) because of the very low midpoint potentials for H_2_ oxidation that are generated by very high pH (Martin, 2022). A summary of energy inputs from serpentinization into the origin of biochemistry is given in Figures 3,4.

## 6. Serpentinization in our solar system

Since olivine is the most abundant silicate mineral that condensed during the formation of the solar system, ultramafic rocks are not unique to Earth and probably ubiquitous in most, if not all, rocky bodies of the solar system (McCollom and Seewald, 2013). Besides Earth there are currently two other solar system bodies where there is evidence for serpentinization: Mars and the Saturn moon Enceladus (Oze and Sharma, 2005; Glein et al., 2015). On Mars ultramafic and serpentinized rocks have been found, with the olivine on Mars having an even higher iron content than that found on Earth (Hoefen et al., 2003; Christensen et al., 2004; Ehlmann et al., 2010; Holm et al., 2015; Tutolo and Tosca, 2023). Organic synthesis has been detected in Martian meteorites that date back 4 Ga which likely happened through serpentinization (McCollom and Seewald, 2013; Steele et al., 2022). Since water (in frozen form) has been detected on the surface of Mars, it is likely that it is also present in Mars’ crust where it could interact with ultramafic material, which means that serpentinization could occur and H_2_ and CH_4_ could be produced (Etiope, 2017). Since both gases would disperse immediately in the Martian atmosphere [which mainly consists of CO_2_ (Mahaffy et al., 2013)], direct monitoring is the only way these gases could be detected (Etiope and Sherwood Lollar, 2013; Holm et al., 2015). Such measurements have been conducted, for example with the Curiosity Rover (House et al., 2022) and on other missions (Lyons et al., 2005) and through observations from Earth (Mumma et al., 2009), however it is unclear if the detection of methane in some of these measurements originates from a contamination in the method employed by the rover (Schoell, 2022).

Using observational data from the Cassini spacecraft, Glein et al. (2015) constructed a model determining the pH and likely chemical composition of Enceladus. They found that Enceladus’ ocean is a Na-Cl-CO_3_ solution with a pH of 11–12. Zolotov (2007) also concluded in a different study that the ocean of Enceladus has a hyperalkaline pH. Enceladus shares the dominance of dissolved Na-Cl with Earth’s oceans but the ubiquity of dissolved Na_2_CO_3_ is more comparable to soda lakes. The high pH is most likely a consequence of serpentinization and after the discovery of H_2_ in the plume of Enceladus (Waite et al., 2017) it is even more likely that serpentinization is still actively occurring. CO_2_ and CH_4_ has also been detected in Enceladus plume and active serpentinization happening would also explain the presence of other organic species in Enceladus plume (Glein et al., 2015; Waite et al., 2017). In a recent study by Postberg et al. (2023) orthophosphates have been found in ice grains of Enceladus plume. The concentrations found indicates that Enceladus ocean has about a 100 times larger abundance of phosphates than Earth’s oceans, with a phosphate: phosphite […] 10:1, since phosphates are more stable under the alkaline conditions of Enceladus ocean. Of the elements considered essential for life (CHNOPS), phosphorus is found the least in astronomical observations and Enceladus is the first ocean world in our Solar System where it has been detected. The availability of phosphorus has been considered a bottle-neck for bio-essential elements on Enceladus and other icy moons and with the discovery of phosphorus in its oceans, Enceladus satisfies yet another requirement for potential habitability.

Other solar system bodies where it is strongly suspected that serpentinization might be happening are other icy moons of the gas giants (Jupiter, Saturn, Neptune, Uranus) (Vance et al., 2007; Schrenk et al., 2013; Vance et al., 2016; Lunine, 2017). Basically, wherever ultramafic rocks get in contact with circulating liquid water with temperatures below 350°C, serpentinization will occur (McCollom and Seewald, 2013). However, unlike with Enceladus, where we are lucky enough to have direct data from the plume because of the Cassini Mission, no direct measurement could be taken yet at these other icy moons and only theoretical models exist (Vance et al., 2007, 2016). It is however likely that we will have data of Europa soon, an icy moon of Jupiter, because missions have been planned from both ESA and NASA, with ESA’s JUICE mission having launched earlier this year (ESA, 2023).

## 7. Conclusion

We return to the passage in the introduction of this paper about meteorite impacts as surface reduction processes vs. reduction in the crust. Available data indicate that the crust can generate reduced nitrogen in serpentinizing systems (Nobu et al., 2023; Shang et al., 2023b) and phosphite during serpentinization (Pasek et al., 2022), with carbon and sulfur reduction reactions being facile under serpentinizing conditions. From that it follows that all of the essential reduction reactions underlying the conversion of the elements on the early Earth from their ancestrally oxidized states (CO_2_, N_2_, HPO_4_^2–^, SO_2_) to their biologically relevant state — reduced C, NH_3_, H_2_S, and in the case of P, the biochemically accessible state — now appear to lie within the range that serpentinizing systems can generate naturally (Russell et al., 2010; Boyd et al., 2020). In traditional cyanide-based RNA-world theories, the reducing functions required at origins are attributed to meteorite impacts or UV-dependent reactions. In subsurface origins theories, serpentinization provides all the reducing power needed for organic synthesis and life. In the subsurface theory, the first organisms were anaerobic chemolithoautotrophs that arose, lived and diversified in complete darkness within the walls of serpentinizing, hence strongly reducing, hydrothermal vents. Such a scenario is much in line with what many microbiologists have thought for many decades. It would also be compatible with the environments presented by a growing number of moons and planets that lie beyond the confines of our atmosphere.

Serpentinization occurs at many sites on Earth and probably elsewhere in our solar system. It is relevant to the study of the origin of life and astrobiology for many reasons outlined here, but mainly because it is a source of a strong and diffusible reductant: H_2_. It generates favorable conditions for organic synthesis and the emergence of life. It synthesizes catalysts that can act as the precursors to enzymes and electron donors that provide energy for metabolism and simple carbon compounds as a nutrient source. Microbial communities of serpentinizing systems, such as Lost City and Old City, provide what could be windows into the physiology of LUCA. The microbial communities of continental and shallow serpentinizing systems like Prony Bay might provide the same kind of window into the physiology of the first microbes as acetogens and methanogens serve as primary producers. Despite the differences between serpentinization sites on land and in the oceans, there is enough overlap between the geochemically produced compounds, the physiological conditions such as the pH and the temperature, as well as the microbial communities, that continental serpentinizing systems can serve as important proxies for the less easily accessible and less abundant deep-sea systems as well as the currently inaccessible serpentinizing systems beyond the confines of Earth.

## Author contributions

LS: Conceptualization, Data curation, Visualization, Writing – original draft, Writing – review & editing. MB, Writing – review & editing. NM: Writing – review & editing. JW, Data curation, Visualization, Writing – review & editing. MP: Data curation, Visualization, Writing – review & editing. WM: Conceptualization, Data curation, Funding acquisition, Project administration, Visualization, Writing – original draft, Writing – review & editing.

## Supplementary material

Supplementary Figure 1 Linear regression between the H_2_ and CH_4_ concentrations in the serpentinizing systems in this study. Here we show a linear regression of hydrogen and methane concentrations of the serpentinizing systems in this study when both compounds were reported or detected. Since mostly ranges of concentrations are reported in Table 1, the maximum concentrations reported were used for the linear regression shown in this figure for simplicity. During the analysis two data points stood out. **(A)** At first there seems to be no relationship between the hydrogen and methane concentrations between the different serpentinizing systems in this study, with r^2^ = 0.0551 and *p* = 0.4626. However, the Prony Bay data reported by (Twing et al., 2022) in Table 4 of their study stands out in contrast to the others and after checking the sources that (Twing et al., 2022) used for Table 4, it seems like they accidentally reported mM amounts of hydrogen and methane for Prony Bay even though the two sources they cite for that data ((Quéméneur et al., 2014) (Postec et al., 2015)) report hydrogen and methane in vol% in their supplementary material. It also contradicts the data reported by (Quéméneur et al., 2023) where the hydrogen and methane concentrations are a magnitude lower, and the ratio of the abundances is also different. **(B)** Linear regression of the serpentinizing systems in this study without the Prony Bay data from (Twing et al., 2022) with r^2^ = 0.0596 and *p* = 0.0136. This already suggests a high significance of the relationship between the concentrations for hydrogen and methane, however Ashadze still seems to be a clear outlier. **(C)** Linear regression of the serpentinizing systems without the data from Prony Bay (Twing et al., 2022) and Ashadze (Schrenk et al., 2013) with r^2^ = 0.8419 and *p* = 0.0002.
