# Supplementary figures and images for: Serpentinization as the source of energy, electrons, organics, catalysts, nutrients and pH gradients for the origin of LUCA and life
Source: Front Microbiol. 2023 Oct 2;14:1257597. doi: 10.3389/fmicb.2023.1257597 (PMC10581274; doi:10.3389/fmicb.2023.1257597)

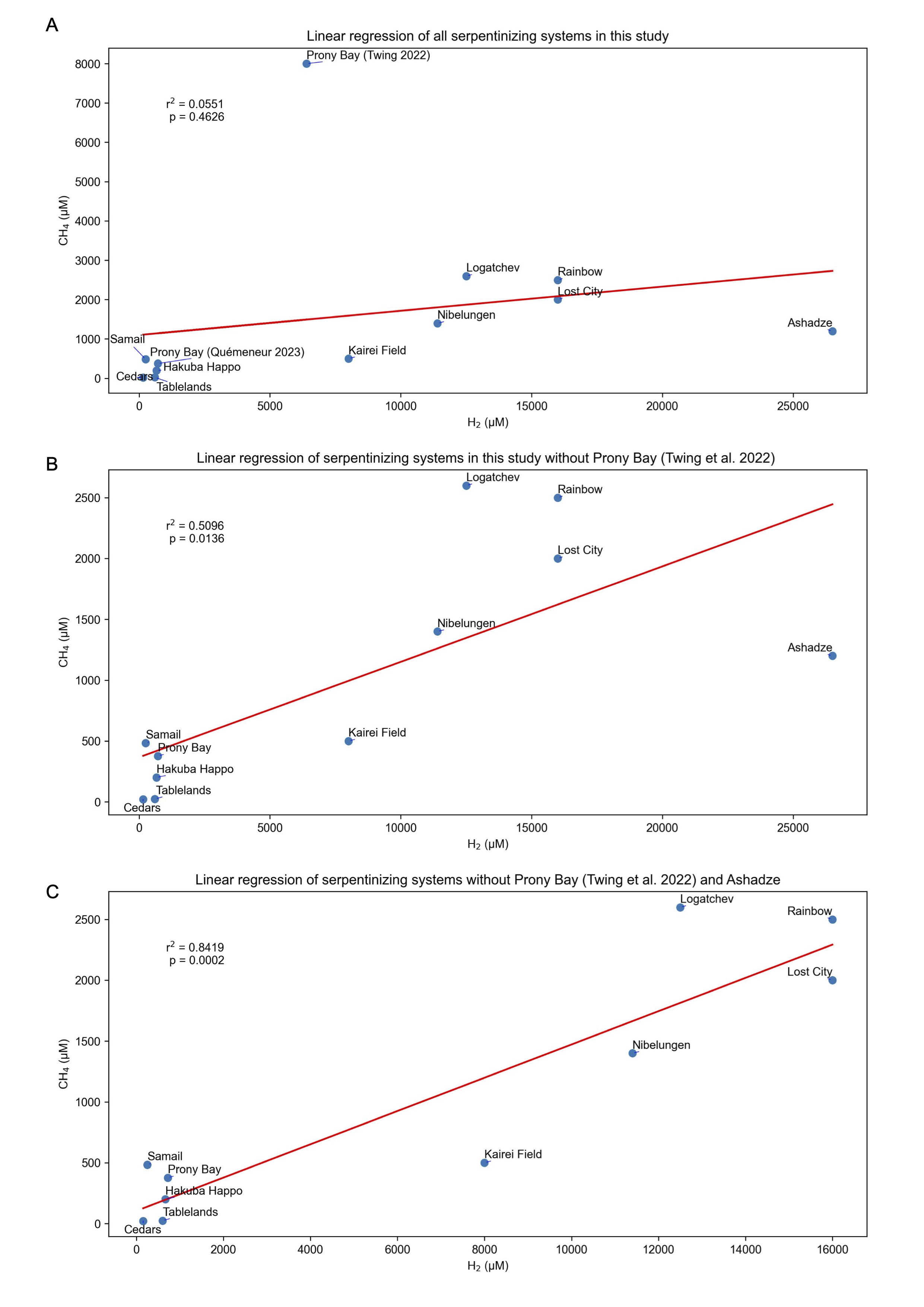

Supplement: Supplementary Figure 1 — Linear regression between the H2 and CH4 concentrations in the serpentinizing systems in this study. Here we show a linear regression of hydrogen and methane concentrations of the serpentinizing systems in this study when both compounds were reported or detected. Since mostly ranges of concentrations are reported in Table 1, the maximum concentrations reported were used for the linear regression shown in this figure for simplicity. During the analysis two data points stood out. (A) At first there seems to be no relationship between the hydrogen and methane concentrations between the different serpentinizing systems in this study, with r2 = 0.0551 and p = 0.4626. However, the Prony Bay data reported by (Twing et al., 2022) in Table 4 of their study stands out in contrast to the others and after checking the sources that (Twing et al., 2022) used for Table 4, it seems like they accidentally reported mM amounts of hydrogen and methane for Prony Bay even though the two sources they cite for that data ((Quéméneur et al., 2014) (Postec et al., 2015)) report hydrogen and methane in vol% in their supplementary material. It also contradicts the data reported by (Quéméneur et al., 2023) where the hydrogen and methane concentrations are a magnitude lower, and the ratio of the abundances is also different. (B) Linear regression of the serpentinizing systems in this study without the Prony Bay data from (Twing et al., 2022) with r2 = 0.0596 and p = 0.0136. This already suggests a high significance of the relationship between the concentrations for hydrogen and methane, however Ashadze still seems to be a clear outlier. (C) Linear regression of the serpentinizing systems without the data from Prony Bay (Twing et al., 2022) and Ashadze (Schrenk et al., 2013) with r2 = 0.8419 and p = 0.0002. [file Image_1.JPEG]
